# Hybrid Antimicrobial Films Containing a Polyoxometalate-Ionic Liquid

**DOI:** 10.1021/acsapm.2c00110

**Published:** 2022-04-12

**Authors:** Ana G. Enderle, Isabel Franco-Castillo, Elena Atrián-Blasco, Rafael Martín-Rapún, Leonardo Lizarraga, María J. Culzoni, Mariela Bollini, Jesús M. de la Fuente, Filomena Silva, Carsten Streb, Scott G. Mitchell

**Affiliations:** † Institute of Inorganic Chemistry I, Ulm University, Albert-Einstein-Allee 11, 89081 Ulm, Germany; ‡ Centro de Investigaciones en Bionanociencias (CIBION), CONICET, Godoy Cruz, 2390, C1425FQD Ciudad de Buenos Aires, Argentina; § Laboratorio de Desarrollo Analítico y Quimiometría (LADAQ), Universidad Nacional del Litoral—CONICET, Ciudad Universitaria, Paraje El Pozo, CC242, S3000 Santa Fe, Argentina; ∥ Instituto de Nanociencia y Materiales de Aragón (INMA-CSIC), Consejo Superior de Investigaciones Científicas-Universidad de Zaragoza, c/Pedro Cerbuna 12, 50009 Zaragoza, Spain; ⊥ CIBER de Bioingeniería, Biomateriales y Nanomedicina, Instituto de Salud Carlos III, 28029 Madrid, Spain; # ARAID—Agencia Aragonesa para la Investigación y el Desarrollo, Av. Ranillas, 1D, 2B, 50018 Zaragoza, Spain; ∇ Facultad de Veterinaria, Universidad de Zaragoza, Calle Miguel Servet 117, 50013 Zaragoza, Spain

**Keywords:** polyoxometalate, polyoxometalate-ionic liquid, guanidinium, antimicrobial, antibiofilm

## Abstract

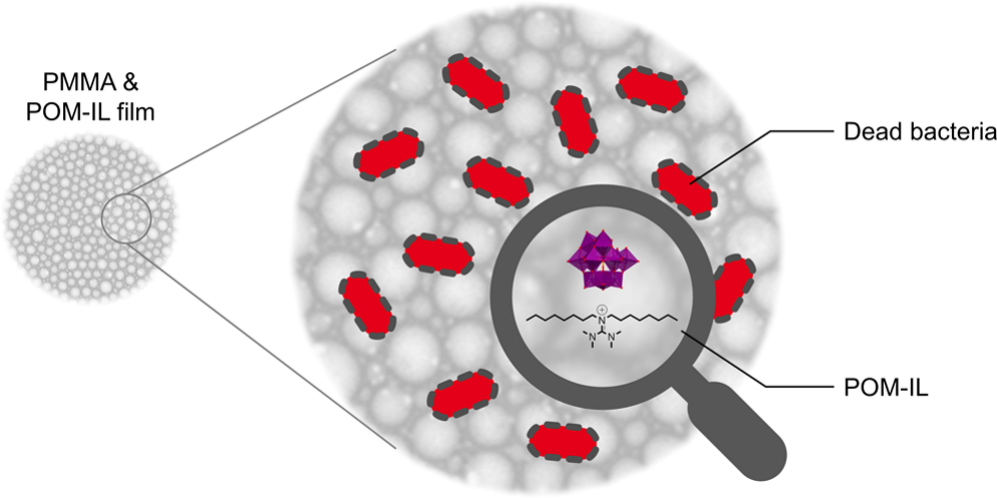

The increasing resistance of pathogenic microorganisms against common treatments requires innovative concepts to prevent infection and avoid long-term microbe viability on commonly used surfaces. Here, we report the preparation of a hybrid antimicrobial material based on the combination of microbiocidal polyoxometalate-ionic liquids (POM-ILs) and a biocompatible polymeric support, which enables the development of surface coatings that prevent microbial adhesion. The composite material is based on an antibacterial and antifungal room-temperature POM-IL composed of guanidinium cations (*N*,*N*,*N*′,*N*′-tetramethyl-*N*″, *N*″-dioctylguanidinum) combined with lacunary Keggin-type polyoxotungstate anions, [α-SiW_11_O_39_]^8–^. Integration of the antimicrobial POM-IL into the biocompatible, flexible, and stable polymer poly(methyl methacrylate) (PMMA) results in processable films, which are suitable as surface coatings or packaging materials to limit the proliferation and spread of pathogenic microorganisms (*e.g.*, on public transport and hospital surfaces, or in ready-to-eat-food packaging).

## 1 Introduction

The development of antimicrobial resistance in pathogens is a global public health challenge. Research efforts worldwide are underway to establish different types of treatments and concepts to prevent humans from contracting (contagious) diseases based on pathogenic microbes such as Methicillin-resistant *Staphylococcus aureus* (MRSA).^1^ Of particular importance in this context is the transfer of microbes between human beings *via* surface contacts, *e*.*g*., door handles, tables, shared utensils, food packaging, *etc*. Thus, there is a great need to develop surface-active antimicrobial coatings, which can be employed in various application scenarios, such as hospitals, care-homes, or communal spaces, *e*.*g*., offices, public offices, transport, *etc*.^2−4^

Recently, ionic liquids and their composites have attracted widespread interest as antimicrobial agents, which could overcome challenges related to acquired antibiotic resistance.^5,6^ Ionic liquids (ILs) are salts with a melting point below 100 °C and are often based on an organic bulky cation and an inorganic anion.^7^ For most applications, room-temperature ILs are the most desirable as they retain their liquidity under typical operating temperatures and can lead to advanced surface coatings. In addition, variation of the cation and anion can be used to target and optimize specific properties including viscosity, solubility, and bioactivity.

Some of us have recently explored polyoxometalate-ionic liquids (POM-ILs) as bioactive surface coatings, where organic ammonium cations are combined with anionic metal oxo clusters, polyoxometalates (POMs) to obtain POM-ILs.^8−10^ The concept was inspired by earlier studies where the synergistic effects of POMs and known antibiotics and organic bioactive compounds have been explored.^11^ In 2006, the activity of several POMs in combination with oxacillin against methicillin- and vancomycin-resistant *S. aureus* was reported, and the authors proposed that the cell proliferation suppression observed was due to synergism between the POM and the oxacillin.^12,13^ In 2017, the synthesis and characterization of POM-based silver(I) phenylethynide compounds with antibacterial and antifungal activities were reported.^14^ The results showed that these compounds have low toxicity in both human and animal cell lines and that their antibacterial and antifungal properties were comparable to those of common antibiotic drugs. In general, the bioactivity of POMs is generally suggested to arise from interactions with amino acids of proteins that lead to biological responses affecting the viability of the bacterial cell.^11^

POM-ILs based on Keggin-type anions ([α-SiW_11_O_39_]^8–^) and tetraalkylammonium ions as active cationic species are reported to be effective antimicrobials against important human pathogens such as *Escherichia coli*, *Pseudomonas aeruginosa*, and especially against the Gram-positive *S. aureus*.^15^ These POM-ILs feature tetra-*n*-heptylammonium or tetra-*n*-octylammonium chains, which interact with the lipid membranes of the bacterial cell. Furthermore, the antibacterial and antifungal activities of POM-ILs were retained, even after loading on silica and deposition as transparent coatings on mineral stone surfaces.^10,16,17^

**Figure 1:**
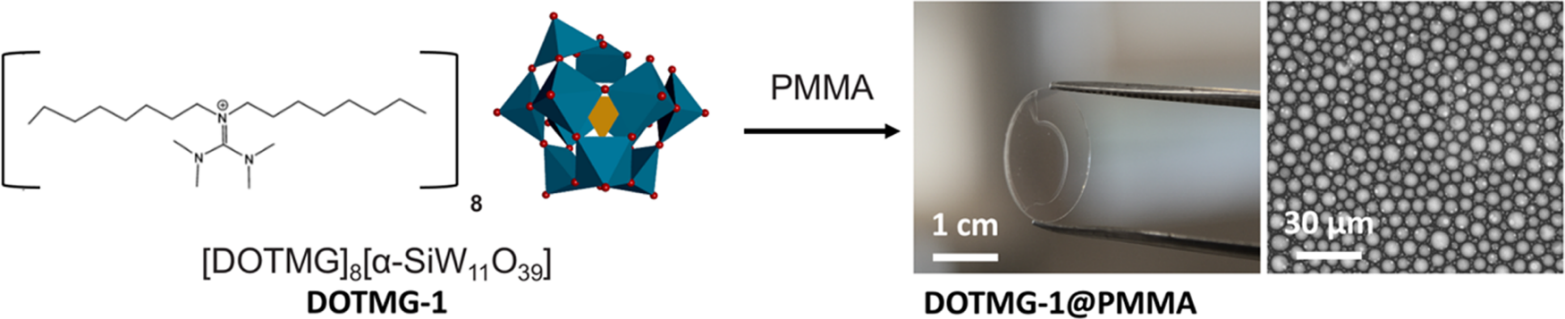
Polyoxometalate-ionic liquid (POM-IL) **DOTMG-1** based on the cation *N*,*N*,*N*′,*N*′-tetramethyl-*N*″,*N*″-dioctylguanidinum (DOTMG) and monolacunary Keggin anion [α-SiW_11_O_39_]^8–^ incorporated into polymethylmethacrylate (PMMA) films (DOTMG-1@PMMA film). Photograph and scanning electron microscopy (SEM) image of transparent Film D, DOTMG-1@PMMA 50/50.

Thus far, the majority of bioactive POM-ILs have focused on alkylammonium cations; therefore, we reasoned that introducing cations with higher antimicrobial efficiency would be a promising route to improve the broad-spectrum antimicrobial activity of the POM-ILs and related materials. In this respect, guanidine derivatives are known to be effective biocides:^18^ the antibiotics streptomycin and chlorhexidine gluconate possess a guanidinium core, while some commercial disinfectants contain dodecyl guanidinium salts.^19^ We proposed incorporating a guanidinium-containing POM-IL into polymeric films could lead to hybrid polymeric materials with broad-spectrum antimicrobial and antibiofilm properties. To this end, here, we report a synthetic route to transform the cation *N*,*N*,*N*′,*N*′-tetramethyl-*N*″,*N*″-dioctylguanidinum (DOTMG) into a POM-IL (**DOTMG-1**) and demonstrate how **DOTMG-1** can be incorporated into a poly(methyl methacrylate) (PMMA) films (Figure 1). The antimicrobial activity of the DOTMG-based POM-IL **DOTMG-1** and resulting polymeric DOTMG-1@PMMA materials against different bacterial and fungal microorganisms reported herein, demonstrate how a new class of bioactive POM-IL composites can be obtained and highlight where further development is required.

## 2 Experimental Section

### 2.1 Instrumentation

Elemental analysis was performed on a Carlo Erba 1108 elemental analyzer. Inductively coupled plasma atomic emission spectroscopy (ICP-AES) was performed on a PerkinElmer Plasma 400 spectrometer. ^1^H and ^13^C NMR spectra were recorded on Bruker Avance 600 (1H: 400.13 MHz; 13C: 100.62 MHz) and Bruker Avance 500 spectrometers (1H: 500.14 MHz, 13C: 125.76 MHz). For the POM, Fourier transform infrared (FT-IR) spectroscopy was performed on a Bruker Vector 22 FTIR instrument. Samples were prepared as KBr pellets. For the films, FT-IR spectroscopy was performed in a Jasco 4700 spectrometer using a magnetic film holder. Signals are given as wavenumbers in cm^–1^ using the following abbreviations: vs = very strong, s = strong, m = medium, w = weak and b = broad. Thermogravimetric analysis (TGA) was performed in a Shimadzu TGA-51 instrument; samples of *ca*. 10 mg were heated at a rate of 10 °C/min under an air atmosphere (flow rate, 30 mL/min) in the range of 30–800 °C. SEM images were acquired using a Phenom Pro scanning electron microscope (SEM). For all images, the working distance was 2.5 ± 0.5 mm. The films were supported on Si wafers and were not sputter-coated. For film A, the study was performed using an accelerating voltage of 5 kV and a charge reduction holder (CRH). These parameters were set to obtain high-quality images of the sample as the film only consisted of PMMA. For films B, C, and D, the study was performed using an accelerating voltage of 5 kV and a standard holder (SH). Atomic force microscopy (AFM) images were acquired using a Bruker multimode 8 SPM (Santa Barbara, CA) with a nanoScope V controller (Santa Barbara, CA). The AFM images were acquired in the intermittent mode using silicon tips doped with antimony, with a spring constant of 42 N/m and a resonance frequency of 320 kHz. Typically, areas of 15 μm × 15 μm were scanned. The image analysis was performed using Gwyddion version 2.46 (Brno, Czech Republic). Average surface roughness (*R*_a_) was determined from AFM height images. For each height image, a reference plane (mean plane) was defined, and a *Z*-axis, perpendicular to that plane, was considered, where *Z* = 0 was on the plane. *Z*-values were calculated from the images in a discrete manner, where *Zj* was defined as the height of the *j*th pixel from the mean plane. Positive *Z*-values are associated with protrusions above the mean plane, while negative *Z*-values are associated with depressions below the plane. The Ra of each AFM image was determined as the average deviation of height values from the mean plane when considering *M* pixels in each image (*M* = 262,144).^20^

### 2.2 Synthesis

All chemicals were purchased from Sigma-Aldrich, ABCR, or Acros Organics and were of reagent grade. The chemicals were used without further purification.

#### 2.2.1 Synthesis of K_8_[α-SiW_11_O_39_]·13H_2_O

The synthesis is a modification of the literature procedure.^21^ Sodium metasilicate (0.50 g, 4.09 mmol) was dissolved at room temperature in 10 mL of distilled water and filtered (solution A). In a 100 mL beaker, sodium tungstate (8.26 g, 25.18 mmol) was dissolved in 3 mL of boiling distilled water (solution B). To the boiling solution B, an aqueous solution of HCl 4 mol/L (8.25 mL) was added dropwise over 5 min with vigorous stirring to dissolve the local precipitate of tungstic acid. Solution A was added, quickly followed by the addition of 2.50 mL of 4 mol/L aqueous hydrochloric acid. The solution was kept boiling for 1 h. After cooling to room temperature, the solution was filtered. KCl (6.80 g, 91.2 mmol) was added to the stirred solution. The resulting white precipitate was collected on a sintered glass funnel (medium porosity), washed with two 20 mL portions of an aqueous KCl solution (1.0 M), then washed with 50 mL of cold water, and finally dried in air (yield: 5.10 g, 1.58 mmol, 69.7% based on Si). FT-IR (cm^–1^): 3420 (b), 2364 (w), 2037 (m), 1624 (m), 995 (s), 917 (s), 888 (vs), 793 (vs), 512 (s), 480 (s). ICP-AES (calculated values within brackets): Si 0.86 (0.87), W 64.31 (62.78).

#### 2.2.2 Synthesis of (C_21_H_46_N_3_)Br (DOTMG-Br)

The synthesis is a modification of the literature procedure.^22^ Tetramethylguanidine (1.15 g, 10 mmol), *n*-octylbromide (1.93 g, 20 mmol), potassium carbonate (1.38 g, 10 mmol), and tetra-*n*-butylammonium bromide (0.06 g, 0.20 mmol) was refluxed in MeCN (40 mL) for 36 h and then cooled to room temperature. Then, the filtrate was collected. *N*-Hexane and water were added, and the mixture was vigorously stirred. The organic layer was separated, and the solvent was evaporated. After removal of solvent traces under vacuum, DOTMG-Br was obtained as a yellow viscous liquid (yield: 0.70 g, 1.66 mmol, 17%). FT-IR (cm^–1^): 3420 (b), 2931 (s), 2848 (m), 2360 (m), 2314 (m), 1600 (s), 1558 (vs), 1458 (m), 1401 (vs), 1375 (m), 1152 (w), 1068 (w), 895 (m), 832 (s), 723 (m). 1H NMR (600 MHz, DMSO-*d*_6_) δ 3.16 (m, 2H, CH_2_N), 3.07 (m, 2H, CH_2_N), 2.89–2.86 (m, 12H, CH_3_N), 1.55 (m, 2H, CH_2_CH_2_N), 1.39 (m, 2H, CH_2_CH_2_N), 1.26 (m, 20H, CH_2_), 0.86 (t, 6 H, *J* = 7.2, CH_3_). 13C NMR (151 MHz, DMSO-*d*_6_) δ 162.4, 48.7, 39.6 (overlapping with DMSO), 31.2, 28.6, 28.5, 27.0, 26.1, 22.0, 13.9 (Figures S1 and S2).

#### 2.2.3 Synthesis of (C_21_H_46_N_3_)_8_[α-SiW_11_O_39_] (**DOTMG-1**)

The synthesis is based on a modified literature procedure.^23,24^ In a round-bottom flask, K_8_[α-SiW_11_O_39_] × 13H_2_O (0.16 g, 0.05 mmol, 1.00 equiv) was dissolved in water (50 mL), and DOTMG-Br (0.4 mmol, 8.00 equiv) was dissolved in dichloromethane (80 mL). The biphasic mixture was vigorously stirred for 15 min. The organic layer was separated, the solvent was removed under reduced pressure, and a light yellow highly viscous liquid was obtained in quantitative yield. *M*_w_ = 5399 gr/mol. FT-IR (cm^–1^): 3450 (b), 2962 (s), 2925 (s), 2853 (m), 2361 (w), 1739 (w), 1595 (s), 1562 (s), 1463 (m), 1407 (m), 1375 (m), 1262 (s), 1097 (s), 1018 (s), 970 (m), 920 (m), 881 (w), 801 (s), 660 (w), 530 (m). EA: % (calculated values in brackets): C 36.6 (37.37), H 7.1 (6.86), N 6.0 (6.22) 1H NMR (600 MHz, DMSO-*d*_6_) δ 3.14 (m, 2H, CH_2_N), 3.09 (m, 2H, CH_2_N), 2.89–2.86 (m, 12H, CH_3_N), 1.55 (m, 2H, CH_2_CH_2_N), 1.38 (m, 2H, CH_2_CH_2_N), 1.24 (m, 20H, CH_2_), 0.85 (t, 6 H, *J* = 7.2, CH_3_). 13C NMR (151 MHz, DMSO-*d*_6_) δ 162.3, 48.8, 39.6, 39.5, 31.2, 28.6, 28.5, 27.0, 26.1, 22.0, 13.9 (Figures S3 and S4). TGA, (in air, 30–800 °C): mass loss observed: 50.2 wt % (observed), 50.5 wt % (calculated) (Figure S5).

#### 2.2.4 Preparation of DOTMG-1@PMMA Films

Silicon wafer substrates (University Wafers) were cleaned with water, ethanol, and air plasma (Zepto, Diener GmbH) before deposition. Four films based on different POM-IL/PMMA weight ratios were prepared: 0/100 (film A), 20/80 (film B), 35/65 (film C), 50/50 (film D). To this end, PMMA (50 mg, *M*_w_ ∼ 350,000) was dissolved in toluene (0.5 mL) and stirred and heated (60–70 °C) for 3 h to have a homogeneous solution of 10% w/v (Solution A). This PMMA solution was mixed with the corresponding amount of **DOTMG-1** and dissolved in toluene to prepare **DOTMG-1** and PMMA blends, which corresponds to 20:80, 35:65, and 50:50 of the mass composition to **DOTMG-1** in PMMA for film B, C, and D, respectively (Table S1).

After shaking for 1 h with an orbital shaker (270 rpm), 20 μL of each solution was deposited by spin-coating onto the center of a squared silicon wafer (15 mm × 15 mm) with the help of a micropipette. For film A (PMMA film), the rate of revolution was 250 rpm for a duration of 5 s, and it was then stepped up to 3200 rpm for a duration of 15 s. For POM-IL composite films, the rate of revolution was set to 300 rpm (A), 330 (B), 360 (C), or 390 (D) for 5 s with an acceleration rate of 120 rpm/s. Then, the rate of revolution was increased to 1200 rpm with an acceleration rate of 500 rpm/s for 15 s. These procedures were set based on the dryness of the films required.

### 2.3 Biological Analysis

#### 2.3.1 Materials

Resazurin sodium salt, sodium phosphate monobasic, sodium phosphate dibasic, sodium cacodylate trihydrate, glutaraldehyde 25%, DMSO, NaCl, and phosphate-buffered saline (PBS) were each purchased from Sigma-Aldrich. All culture media were purchased from Scharlab, S.L. (Spain). For the bacterial assays, the following culture media were used: Tryptone Soy Agar (TSA), Mueller Hinton Agar supplemented with chloramphenicol (MHAclo) and brain heart infusion agar (BHIA) as solid media; Luria-Bertani (LB) and nutrient broth (NB) as liquid media. For the fungal assays, the following culture media were used: Sabouraud dextrose agar supplemented (SDA) with chloramphenicol, yeast malt agar (YMA), and potato dextrose agar (PDA) as solid media; RPMI 1640 (with l-glutamine and a pH indicator, without bicarbonate) supplemented with glucose to a final concentration of 2% and 3-(*N*-morpholino) propanesulfonic acid (MOPS) at a final concentration of 0.165 mol/L, pH 7.0, yeast malt broth (YMB) and malt dextrin peptone (MEP) as liquid media.

#### 2.3.2 Microorganisms and Growth Conditions

Four bacterial strains were tested in the antibacterial assays: *E. coli* DH5α and *E. coli* O157:H7 (verotoxigenic *E. coli* (VTEC)) CECT 5947 as Gram-negative models, *Bacillus subtilis* 1904-E and *Listeria monocytogenes* CECT 911 as Gram-positive models. Four molds from the colección española de cultivos tipo (CECT) were tested in the antifungal assays: *Aspergillus niger* CECT 2088, *Cladosporium cladosporioides* CECT 2111, *Aspergillus ochraceus* CECT 2093, and *Penicillium expansum* CECT 2275. Fungal spore suspensions were stored in 0.1% Tween and 20% glycerol at −80 °C prior to use. All bacterial and fungal growth conditions are summarized in Table S2. Briefly, all bacteria were incubated for 24 h at 37 °C, LB was the selected liquid media for the Gram-negative strains, and NB was used for the Gram-positive strains. Three different solid media were used for the bacterial cultures, TSA for *E. coli* DH5α and *B. subtilis*, MHA for verotoxigenic *E. coli,* and BHIA for *L. monocytogenes*. All of the fungal strains were incubated for 4 days to prepare the inoculum at 35 °C for *A. niger* and at 25 °C for *A. ochraceus*, *C. cladosporioides*, and *Paspalum expansum*. RPMI was used as liquid media for *A. niger* and *C. cladosporioides*, MEP for *A. ochraceus*, and YMB for *P. expansum*. Three different solid media were used for mold incubation, SDA for *A. niger* and *C. cladosporioides*, YMA for *A. ochraceus*, and PDA for *P. expansum*.

#### 2.3.3 Cytotoxicity Assays

Cytotoxicity assays were performed using CellTiter-Glo Luminescent Cell Viability Assay (Promega), a CO_2_ incubator steri-cult 3311 (Thermo Scientific), and Orion II microplate luminometer (Titertek-Herthold, Germany), according to the manufacturer’s instructions. The cell lines used were HEK293T (human embryonic kidney cell line, used for transient transfection) obtained via the ATCC (CRL-3216TM), and TZM-bl (HeLa cell derivative used as reporter cell line for human immunodeficiency virus (HIV) infection) obtained via the National Institute of Health (NIH) Acquired Immune Deficiency Syndrome (AIDS) reagent program (catalogue number 8129). To assess the potential cytotoxic activity, 100 mg of **DOTMG-1** and DOTMG-Br was initially dissolved in 1 mL of dimethyl sulfoxide (DMSO). This solution was then diluted 1:200 into PBS, to avoid any DMSO-related cytotoxic effect. HEK293T and TZM-bl cells were seeded into a 96-well plate in 100 μL of Dulbecco’s modified Eagle’s medium (DMEM) to have 30–40% of cells confluency at the time of seeding. The next day, the cells were incubated with a serial dilution of **DOTMG-1** and DOTMG-Br, with DMSO as the only negative control and Triton-X100 as a positive control for cell death. The cells were incubated for 2 days at 37 °C, and cell viability was assessed with the CellTiter-Glo assay by measuring the total amount of adenosine triphosphate (ATP). Determination of the cytotoxic concentration 50 (CC50) was done using the log(inhibitor) *vs* response-variable slope (four parameters) nonlinear regression option available in the GraphPad Prism 7.03 software (La Jolla, California).

#### 2.3.4 Microbial Growth Inhibition Assay in the Presence of **DOTMG-1**

Bacterial and fungal growth was recorded by measuring the optical density (OD) of the samples at 620 nm over a 24 h period using a microplate reader (Thermo Scientific MULTISKAN GO). Results were compared with the OD variation of a positive control culture containing only bacteria or fungi and a negative control containing the compound in culture media. All control and antibacterial assays were replicated in sextuplet to calculate the mean values and standard deviations, and each experiment was repeated on four separate occasions to verify the reproducibility of the results. The modal value was chosen as the minimum inhibitory concentration (MIC).

##### 2.3.4.1 Bacterial Growth Inhibition Assay

The minimum inhibitory concentration (MIC) of the POM-IL **DOTMG-1** was determined against the four bacterial strains (*E. coli* DH5α, VTEC, *B. subtilis,* and *L. monocytogenes*). The bacteria were thawed and incubated in the appropriated solid culture media for 24 h at 37 °C. An inoculum of 10^7^ CFU/mL was prepared in the appropriate liquid media, and 100 μL was added to a 96-well plate containing 98 μL of the appropriate liquid media and 2 μL of the POM-IL dissolved in DMSO at the desired concentration (500, 250, 125, 62.5, 31.25, 15.62, 7.81, 3.91, 1.95, and 0.98 μg/mL). Positive controls contained bacteria and liquid media, while the negative controls contained only **DOTMG-1** dissolved in culture media. The bacterial growth curves were recorded over a 24 h period by measuring the optical density (OD) of the samples at 620 nm. Results were compared with the OD variation of a control culture containing bacteria without POM-IL.

##### 2.3.4.2 Bacterial Cell Viability Assay

Bacterial cell viability after the treatment with **DOTMG-1** was analyzed using a Resazurin (7-Hydroxy-3H-phenoxazin-3-one 10-oxide) assay. A 10^7^ CFU/mL bacterial inoculum was incubated with different concentrations of **DOTMG-1** as in the bacterial growth inhibition assay (Section 2.3.4.1) on a 96-well plate. A positive (bacteria and liquid media) and a negative control (liquid media and POM-IL) were also included. After the incubation of the plates for 24 h at 37 °C, 0.1 mg/mL Resazurin (dissolved in LB for *E. coli* DH5α and VTEC and in NB for *B. subtilis* and *L. monocytogenes*) was added into each well and incubated at 37 °C in the dark for 1 h under stirring. The Resazurin compound (blue) turns pink in the presence of viable microorganisms as a result of their metabolic activity; therefore, pink wells after the incubation time will indicate live, viable bacteria, while blue wells indicate a loss of metabolic activity, which is one of the first cascade events in the mechanism of cell death. The bacterial viability was also confirmed by subculturing 10 μL of each well on solid media. After incubating the plates for 24 h at 37 °C, the MBC values obtained with Resazurin were compared with the lack of colonies on the solid media.

##### 2.3.4.3 Fungal Growth Inhibition Assay

The determination of the minimum inhibitory concentration (MIC) of the POM-IL **DOTMG-1** against the four fungal strains (*A. niger*, *A. ochraceus*, *C. cladosporioides,* and *P. expansum*) was performed using a broth microdilution method according to the European Committee on Antimicrobial Susceptibility Testing Guidelines (E.DEF 9.3.1).^25^ Fungi spores were incubated for 5 days in solid media at the corresponding temperature for each mold. The aerial part of the fungi was recovered with a swab and resuspended in distilled sterile water with 0.1% tween to obtain a suspension of 10^6^ conidia/mL. This suspension was then diluted to 10^5^ conidia/mL in distilled sterile water. To determine the MIC, **DOTMG-1** was dissolved in DMSO at the desired concentrations (2000, 1000, 500, 250, 125, 62.5, 31.25, 15.625 μg/mL) and 2 μL of each solution was added to each well of a 96-well plate containing 98 μL of culture media and 100 μL of the 10^5^ conidia/mL suspension. The positive control contained only fungal spores and culture media, while the negative control contained only **DOTMG-1** dissolved in culture media. After an incubation period of 48 h, at 35 °C for *A. niger* and at 25 °C for *A. ochraceus*, *C. cladosporioides*, and *P. expansum*, according to CECT recommendations, the minimum inhibitory concentration (MIC) values were determined as the lowest POM-IL concentration able to inhibit fungal growth visible to the naked eye. The results were confirmed by measuring the optical density (OD) of the samples at 620 nm and compared with the OD of the positive control (only bacteria without POM-IL).

#### 2.3.5 Surface Antimicrobial Activity

The surface antimicrobial activity of the POM-IL **DOTMG-1** was studied using a modified JIS Z 2801 standard (Reference number: JIS Z 2801: 2000 (E); ICS 07.100.10; 11.100); 2 cm × 2 cm sterilized glass slides were coated with **DOTMG-1**, dissolved in acetone, at different concentrations (1, 2, 4, 8, and 16 μg/cm^2^). The coated slides were dried under ultraviolet (UV) light for 20 min to avoid any external contamination and then 50 μL of a 10^7^ CFU/mL bacterial suspension were added over the coated slides and over a control slide without coating. A coverslip was put on top of each sample to ensure comparable contact surfaces. The samples were incubated at 37 °C for 24 h in a humidity chamber, and after the incubation time, the bacteria were extracted by vortexing the samples inside a 50 mL falcon tube with 20 mL of liquid media for 1 min. The liquid media containing the extracted bacteria was then diluted and sown on the appropriate solid media for each microorganism. The colonies grown on the plates were counted after incubation at 37 °C for 24 h. The percentage of bacterial growth reduction was obtained by comparing the number of colonies present in the plates from the coated samples and the colonies present in the plates from the control sample.

#### 2.3.6 Electron Microscopy Studies on Microbial Response to **DOTMG-1** and DOTMG-1@PMMA

Transmission electron microscopy (TEM) images were acquired by depositing fixed bacteria on a TEM grid and visualizing the samples using bright-field imaging in an FEI Tecnai T20 microscope operating at 200 kV. Environmental scanning electron microscopy (ESEM) data were collected on a Quanta FEG-250 (FEI Company) field emission SEM for high-resolution imaging working in ESEM mode using a gaseous secondary electron detector (GSED) under high relative humidity conditions.

##### 2.3.6.1 TEM Analysis of the Bacteria Incubated with **DOTMG-1**

Bacterial morphology after the treatment with the POM-IL **DOTMG-1** was studied by TEM. A 10^7^ CFU/mL bacterial suspension of each bacterium (*E. coli* DH5α, VTEC, *B. subtilis,* and *L. monocytogenes*) was incubated with the compound at **DOTMG-1** concentrations corresponding to the MIC and 1/2 MIC values for each of them. The assay was performed on a 12-well plate, where each well contained 1 mL of the bacteria suspension, 980 μL of liquid media, and 20 μL of the compound at the corresponding concentration. Then, the plate was incubated at 37 °C for 24 h with agitation in an incubator. Fixation of these bacterial suspensions was carried out prior to TEM analysis to preserve the biological sample. The samples were centrifuged at 3000 rpm for 15 min, and then the pellet was resuspended into 1.5 mL of sterile PBS. Another centrifugation at 3000 rpm for 15 min was carried out and the pellet containing the bacteria was resuspended in 1.5 mL of 2.5% glutaraldehyde in phosphate buffer 10 mM at pH 7.2 for fixation of the cells. The samples were incubated for 2 h on a Ferris wheel and then washed once with 1 mL of sterile phosphate-buffered saline (PBS) at pH 7.4 and three times with sterile distilled water with centrifugations at 3000 rpm for 15 min between washes for cell recovery and to remove excess glutaraldehyde. The pellets were resuspended in 1.5 mL of sterile Milli-Q water and kept at 4 °C for further analysis. Each sample (2 μL) was deposited on a carbon-coated copper grid (Cu200 mesh) and left to dry at room temperature overnight. TEM images were obtained in a TECNAI T20 electron microscope (FEI) operating at 60 kV.

##### 2.3.6.2 ESEM Visualization of the Molds Incubated with the **DOTMG-1**

The effect of the POM-IL on fungal cells was also studied by environmental scanning electron microscopy (ESEM), which avoids the need for cell fixation. First, a suspension of 10^5^ conidia/mL was prepared as described in Section 2.3.4.3. This suspension (1 mL) was then incubated with the compound at concentrations corresponding to the MIC and 1/2 MIC values for each mold, by adding 20 μL of **DOTMG-1** DMSO solution (100× the desired final **DOTMG-1** concentration) and 980 μL of liquid media to ensure a maximum DMSO concentration of 2% in the culture medium. The solution was incubated on a glass vial for 48 h at the corresponding temperature for each mold. After the incubation time, the samples were centrifuged at 10,000 rpm for 10 min and the supernatant was discarded. The pellet, containing the mold, was resuspended into 1 mL of saline and filtered through a sterile polycarbonate membrane (13 mm diameter, pore size 0.22 μm). Afterward, the membranes containing the molds were placed on a 12-well plate and fixed. The fixation protocol was performed as follows: the membranes were washed once with 2 mL of saline and then fixed with 2 mL of cacodylate buffer 0.1 M at 37 °C for 90 min. To dehydrate the molds, increasing concentrations of methanol were used (5 min with methanol 30%, 5 min with methanol 50%, 5 min with methanol 70%, 10 min with methanol 100%, and 5 min with methanol 100%). The fixed samples were visualized on a Quanta FEG-250 (FEI Company) field emission SEM for high-resolution imaging operating in ESEM mode using a GSED detector.

#### 2.3.7 Biological Performance of the Films

The antibacterial activity of the four different films (A, B, C, and D) was tested against Gram-positive *B. subtilis* and *L. monocytogenes* as well as Gram-negative *E. coli* DH5α. Films were placed in a six-well plate, inoculated with 50 μL of a 10^6^ CFU/mL inoculum of the different bacteria, and incubated at 37 °C for 4 h in a humidity chamber. After the incubation time, the films were rinsed with 1 mL of saline, which was then diluted and plated on TSA plates. The films were rinsed again with 1 mL of distilled sterile water to remove any remaining salts and visualized using ESEM.

##### 2.3.7.1 Environmental Scanning Electron Microscopy (ESEM) Visualization of the Inoculated Films

The effect of the film on the bacteria was also studied by ESEM. The films (after being rinsed) were visualized on a Quanta FEG-250 (FEI Company) field emission SEM for high-resolution imaging working in ESEM mode using a GSED detector. Note that due to the pathogenicity of *L. monocytogenes*, these samples required fixation with glutaraldehyde. Unfortunately, during the fixation process and subsequent washing protocols, the bacteria were removed repeatedly from the silicon wafer surface, and despite a number of attempts, these issues could not be resolved.

## 3 Results and Discussion

### 3.1 Synthesis and Characterization of **DOTMG-1**

The POM-IL **DOTMG-1** was synthesized by a modification of a previously reported cation metathesis route and was obtained in quantitative yield. The sample composition and purity were confirmed by NMR and FT-IR spectroscopies as well as elemental analyses (refer to the Supporting Information for further details).

### 3.2 Preparation of DOTMG-1@PMMA Films

DOTMG-1@PMMA films were obtained by preparing a solution of **DOTMG-1** and PMMA in toluene and spin-coating these solutions on silicon wafers as substrates. The hybrid films were obtained by air-drying the samples; for more details, see Section 2.2.4. Herein, four hybrid DOTMG-1@PMMA films are discussed in detail, varying in their POM-IL/PMMA weight ratio: film A (0/100, POM-IL/ PMMA), film B (20/80), film C (35/65), film D (50/50).

### 3.3 Characterization of DOTMG-1@PMMA Films

**Figure 2:**
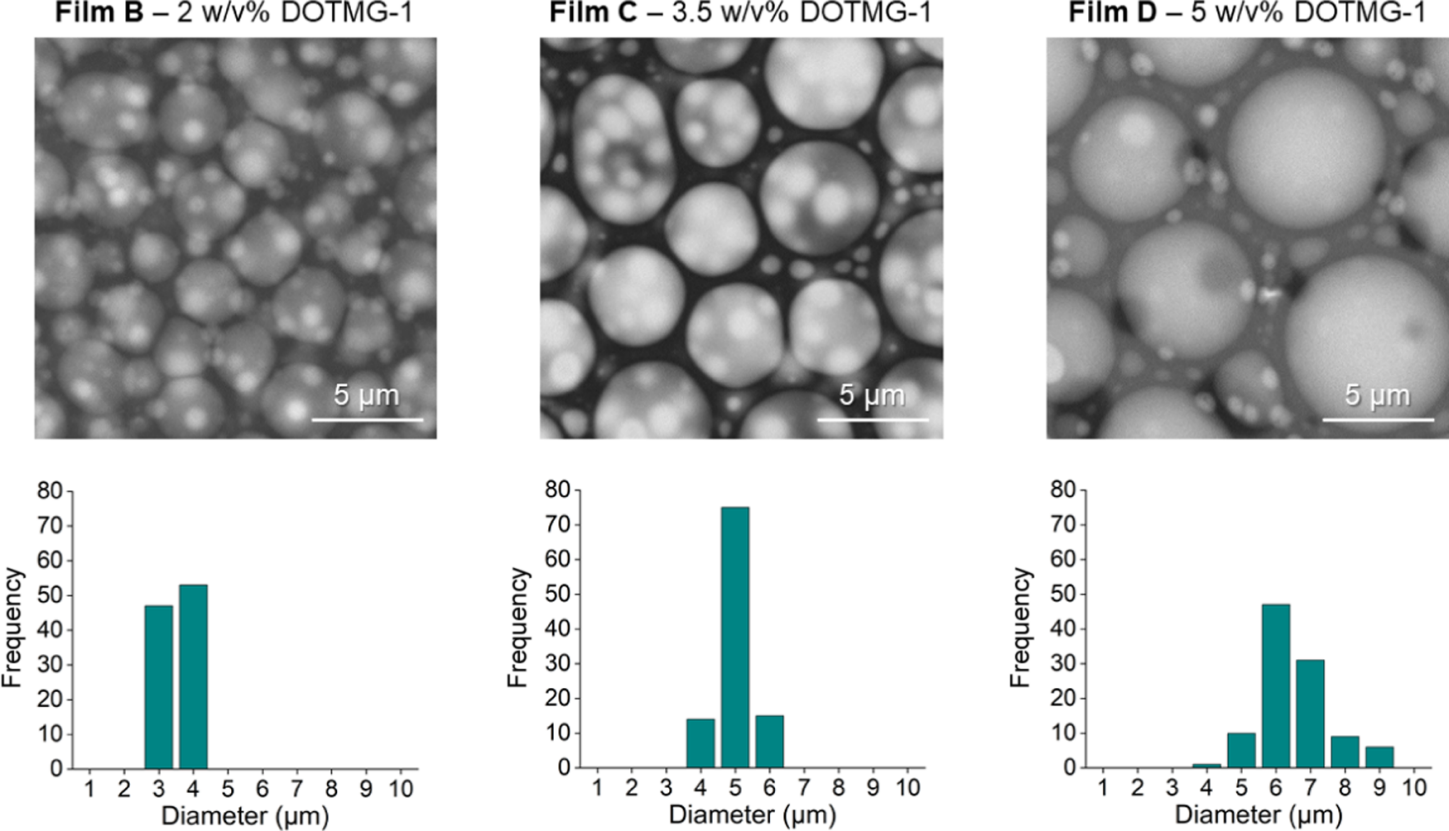
SEM micrographs of the films DOTMG-1@PMMA films B, C, and D prepared with 2, 3.5, and 5 w/v % **DOTMG-1**, respectively. Scale bar = 5 μm.

The chemical makeup of the films was characterized by FT-IR spectroscopy, which confirmed the presence of the POM, DOTMG, and PMMA signals (see Figure S5 for details). SEM analysis of the films shows that film A (purely PMMA-based) has a smooth surface and has a homogeneous structure (Figure S6). In contrast, films B, C, and D (2, 3.5, and 5 w/v % **DOTMG-1**, respectively) show a rough surface structure and a biphasic composition, which we assign to (partial) phase separation during the drying process (Figures S7–S9). SEM data also show that increasing the POM-IL content in the films also increases the size of the spherical structures embedded within the polymeric matrix. The diameter of these structures is equal to 3.05 ± 0.30 nm in film B (2 w/v %) and increases to 4.40 ± 0.52 nm in film C (3.5 w/v %) and to 5.85 ± 0.95 nm in film D (5 w/v %) (Figure 2). We attribute this larger and more “relaxed” droplet size to the fact that the **DOTMG-1** was less confined by the polymeric matrix at higher POM-IL:PMMA concentration ratios.

**Figure 3:**
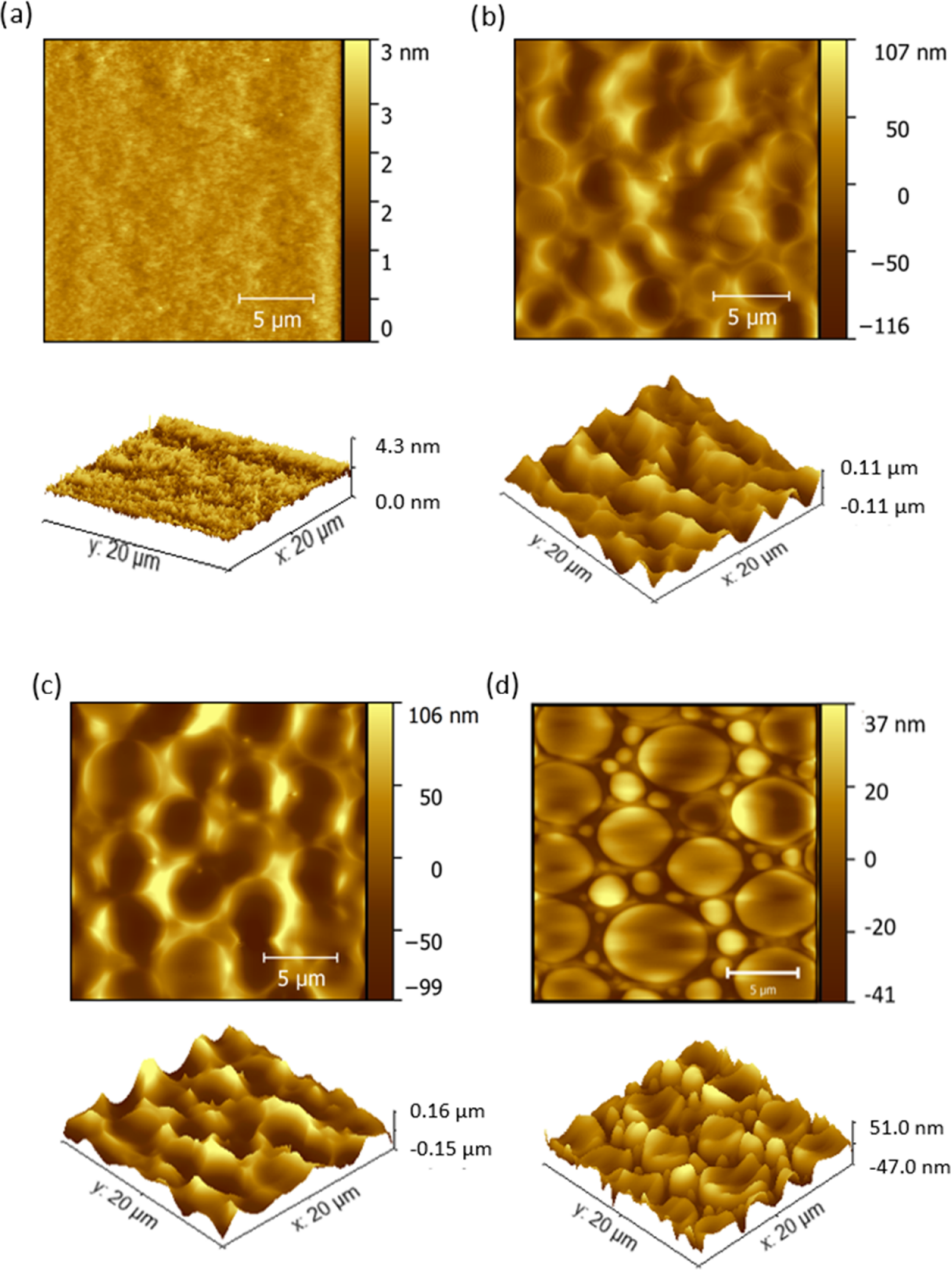
AFM images of POM-DOTMG-1 films prepared (a) without POM-IL **DOTMG-1** and with (b) 2 w/v %, (c) 3.5 w/v %, and (d) 5 w/v % **DOTMG-1**, respectively.

Atomic force microscopy (AFM) was used to further probe the DOTMG-1@PMMA films and verified that increasing the POM-IL content in the films increased the size of the POM-IL assemblies embedded within the PMMA matrix (Figure 3). For further AFM data of films B, C, and D, including characterization of the surface roughness, refer to Figures S10–S13 in the Supporting Information.

### 3.4 Cytotoxicity of **DOTMG-1**

The cytotoxicity and cell viability assays were performed to study the toxic effect of the compounds on the cells. The concentration-dependent cytotoxic effect of **DOTMG-1** and DOTMG-Br, along with a positive and negative control (Triton-X100 and DMSO, respectively), was measured in HEK293T cells and TZM-bl cells (Figures S14 and S15). Cell viability decreases as the concentration of the POM-ILs increases. As can be seen in Table 1, according to the calculated CC_50_ for the ILs in both cell lines, the formation of the POM-IL **DOTMG-1** decreases the cytotoxicity of the precursor DOTMG-Br (as seen by the μg/mL data in Table 1), highlighting that the combination of organo-cation and POM anion leads to improved biocompatibility, which is likely due to the reduction in the aqueous solubility of the POM-IL.

**Table 1:** CC_50_ Values of **DOTMG-1** and DOTMG-Br

compounds	CC_50_ (μg/mL)		CC_50_ (μM)	
	HEK293T cells	TZM-bl cells	HEK293T cells	TZM-bl cells
DOTMG-Br	2.46	0.59	5.85	1.40
DOTMG-1	14.20	2.72	2.63	0.50

### 3.5 Antimicrobial Activity of **DOTMG-1**

#### 3.5.1 Microbial Growth Inhibition in the Presence of **DOTMG-1**

##### 3.5.1.1 Bacterial and Fungal Growth Inhibition Assay

The antimicrobial activity of **DOTMG-1** was first studied by determining the minimum inhibitory concentration (MIC) of the compound against four bacterial strains (*E. coli* DH5α, VTEC, *B. subtilis,* and *L. monocytogenes*) and against four fungal strains (*A. niger*, *A. ochraceus*, *C. cladosporioides,* and *P. expansum*). **DOTMG-1** exhibited antimicrobial activity against all of the bacterial and fungal strains at low concentrations (from 1.95 μg/mL for the most sensitive bacterium to 250 μg/mL for the most resistant mold; see Table 2). The Gram-positive bacterial cell wall is composed of a thick, multilayered peptidoglycan sheath outside of the cytoplasmic membrane, while the Gram-negative cell wall is composed of an outer membrane linked by lipoproteins to thin, mainly single-layered peptidoglycan. The peptidoglycan is located within the periplasmic space that is created between the outer and inner membranes. It therefore follows that the most sensitive microorganism was Gram-positive *B. subtilis*, with a MIC value of 1.95 μg/mL, while the MIC of **DOTMG-1** against pathogenic bacteria *L. monocytogenes* was equal to 31.25 μg/mL. The Gram-negative bacteria *E. coli* DH5α and VTEC were more resistant to **DOTMG-1**, both possessed MICs corresponding to 125 μg/mL. Such differences in MIC between the Gram-negative and Gram-positive bacteria suggest that the mechanism of action of the **DOTMG-1** may involve damage to the cell membrane. The MIC of **DOTMG-1** against molds *C. cladosporioides* and *P. expansum* corresponded to 31.25 μg/mL, while *A. ochraceus* equaled 125 μg/mL. As expected, the pervasive and resistant mold *A. niger* was the most resilient of the assayed microorganisms against **DOTMG-1**, but even so, the MIC was found to be low, at just 250 μg/mL. All of the corresponding MIC values are summarized in Table 2.

**Table 2:** Minimum Inhibitory Concentration (MIC) and Minimum Bactericidal Concentration (MBC) of **DOTMG-1** against Different Microorganisms^a^

microorganism		MIC (μg/mL)	MIC (μM)	MBC (μg/mL)
Gram-negative bacteria	*E. coli* DH5α	125	23.15	125
	VTEC	125	23.15	125
Gram-positive bacteria	*B. subtilis*	1.95	0.36	3.91
	*L. monocytogenes*	31.25	5.78	31.25
molds	*A. niger*	250	46.30	ND
	*A. ochraceus*	125	23.15	ND
	*C. cladosporioides*	31.25	5.78	ND
	*P. expansum*	31.25	5.78	ND

a ND: Not determined.

Bacterial cell growth in the presence of the antimicrobial **DOTMG-1** was monitored *via* the optical density of the bacterial culture containing increasing **DOTMG-1** concentrations. Figure S17 shows how the bacterial growth was inhibited for concentrations corresponding to the MIC value and higher, while the cultures containing the compound at concentrations below the MIC presented a growth curve similar to the negative control sample containing only bacteria in the growth medium.

##### 3.5.1.2 Bacterial Cell Viability Assay

The minimum bactericidal concentration (MBC) of **DOTMG-1** against the four bacterial strains was obtained by a Resazurin cell viability assay (Figure S18) and confirmed by subculturing in solid culture media. The results obtained confirmed that the MBC values of **DOTMG-1** against *E. coli* DH5α, VTEC, and *L. monocytogenes* were commensurate with the MIC values: 125, 125, and 31.25 μg/mL, respectively (Table 2). On the other hand, the MBC for *B. subtilis* (3.91 μg/mL) was 2 times higher than its MIC value (1.95 μg/mL) (Table 2). Importantly, these data confirm that **DOTMG-1** POM-IL is not only inhibiting bacterial cell growth but is also killing bacterial cells.

#### 3.5.2 Surface Antimicrobial Activity of **DOTMG-1**

The surface antimicrobial activity of **DOTMG-1**, which can be related to its ability to prevent bacterial adhesion and, consequently, biofilm formation of the four bacterial strains (*E. coli* DH5α, VTEC, *B. subtilis,* and *L. monocytogenes*), was studied using a modified JIS Z 2801 standard. To obtain the percentage of bacterial reduction, the number of colonies counted on the plates from the POM-IL **DOTMG-1**-coated samples were compared with the colonies counted on the plates from the control samples without coating. In agreement with the MIC/MBC results, we observed a 100% bacterial growth reduction in Gram-positive bacteria at lower concentrations of **DOTMG-1** than for the Gram-negative *E. coli* strains. **DOTMG-1** achieved a 100% bacterial cell reduction at low concentrations ranging from 2 to 8 μg/cm^2^ (Table 3).

**Table 3:** Concentration of **DOTMG-1** Surface Coating (in μg/cm^2^) Required for 100% Bacterial Reduction

microorganism	DOTMG-1 (100% bacterial reduction)
*E. coli* DH5α	8 μg/cm^2^
VTEC	4 μg/cm^2^
*B. subtilis*	2 μg/cm^2^
*L. monocytogenes*	2 μg/cm^2^

#### 3.5.3 Electron Microscopy Studies on the Microbial Response to **DOTMG-1**

##### 3.5.3.1 TEM Analysis of the Bacteria Incubated with **DOTMG-1**

**Figure 4:**
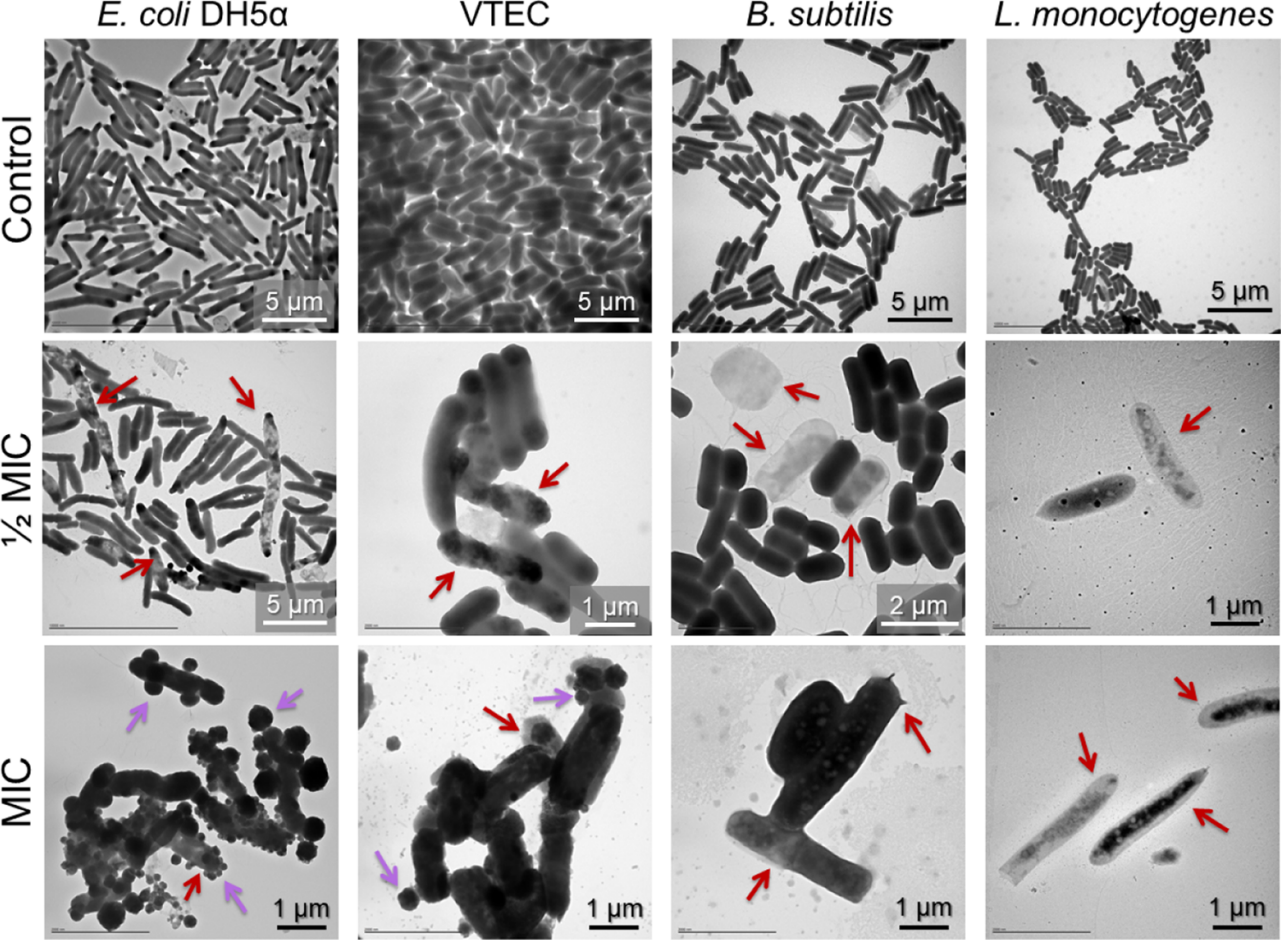
TEM images of the bacterial response to **DOTMG-1** at concentrations corresponding to its 1/2 MIC and MIC for each bacterial strain (*E. coli* DH5α, VTEC, *B. subtilis,* and *L. monocytogenes*) as well as TEM images of these bacteria in the absence of **DOTMG-1** (control). At a sub-MIC concentration, bacteria are still able to reproduce, but cell damage is already observed (red arrows). At MIC concentration, bacterial cells are no longer capable of dividing, and most bacteria show cell damage and alterations in their morphology (red arrows). At MIC concentration, both Gram-negative bacteria (*E. coli* DH5α and VTEC) are covered by POM-IL aggregates (purple arrows), probably due to interaction between the compound and the characteristic outer cell membrane of these bacteria.

Transmission electron microscopy (TEM) was performed on samples of *E. coli* DH5α, VTEC, *B. subtilis,* and *L. monocytogenes*, to study the morphology of the bacterial cells incubated with **DOTMG-1** at concentrations corresponding to its MIC and 1/2 MIC for each of the bacterial strains. At sub-MIC concentrations (1/2 MIC), although all bacterial strains displayed indications of replication, there were several signs of stress. Notably, *E. coli* DH5α, *B. subtilis,* and *L. monocytogenes* cultures showed signs of damage to the cell wall and there was an appreciable accumulation of the cytoplasmic content in the cell ends in the case of VTEC (Figure 4). At concentrations corresponding to the MIC value, bacterial cell growth for the four bacterial strains appeared to be completely inhibited and cells displayed a loss of their structural integrity. Both Gram-negative bacterial cells (*E. coli* DH5α and VTEC) were completely covered by POM-IL aggregates, probably due to the outer cell membrane containing lipopolysaccharides, which also could protect them against the **DOTMG-1** compound, explaining the higher MIC values of these strains (Figure 4).

##### 3.5.3.2 ESEM Visualization of the Molds Incubated with **DOTMG-1**

**Figure 5:**
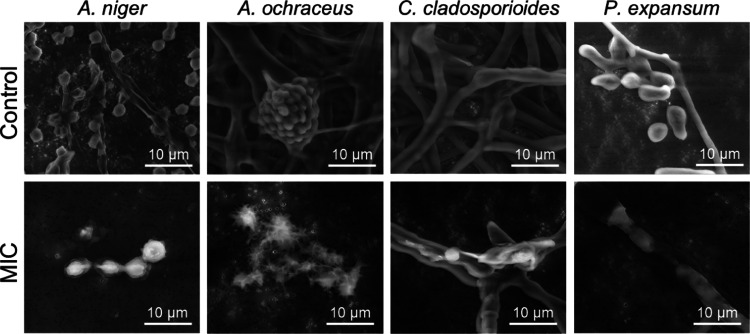
ESEM images of the four molds (*A. niger*, *A. ochraceus*, *C. cladosporioides,* and *P. expansum*) in the absence of antimicrobial **DOTMG-1** (control) and inoculated with **DOTMG-1** at the corresponding MIC concentration for each fungal strain.

The effect of the POM-IL on mold morphology was studied by incubating each of the four molds (*A. niger*, *A. ochraceus*, *C. cladosporioides,* and *P. expansum*) with **DOTMG-1** at the MIC concentration and 1/2 MIC, and then analyzing the samples by environmental scanning electron microscopy (ESEM). At both MIC and 1/2 MIC concentrations, mold structure was affected. Additionally, there was noticeably less sporulation, damage to the fungal hyphae and conidia, including morphological alterations (Figure 5).

#### 3.5.4 Biological Performance of the DOTMG-1@PMMA Films

**DOTMG-1** was embedded in a PMMA matrix to obtain antimicrobial and antibiofilm surfaces. The antimicrobial effect of the hybrid DOTMG-1@PMMA films against *E. coli* DH5α, *B. subtilis,* and *L. monocytogenes* was studied by inoculating the films with a bacterial culture, incubating them for 4 h, and plating the culture that was isolated from the rinsing of the samples. As expected, for the control film (*A* = 100% PMMA; no **DOTMG-1**), the bacterial concentration remained constant at ∼10^6^ CFU/mL following the 4 h incubation period. On the other hand, the hybrid DOTMG-1@PMMA films containing the **DOTMG-1** POM-IL at 2, 3.5, and 5 w/v % (B, C, and D, respectively) all displayed a 100% reduction for each of the three bacteria that were assayed. To infer the mechanism of action, the material–microbe interactions in the hybrid DOTMG-1@PMMA films incubated with nonpathogenic *E. coli* DH5α and *B. subtilis* were studied by ESEM (Figure S19). Due to the high antimicrobial and antibiofilm activity displayed by the DOTMG-1@PMMA films, very few bacterial cells were observed on the surface of the samples, indicating low adhesion and/or death of the bacterial cells that come into contact with the polymer surface. Unfortunately, due to the low number of cells adhering to the DOTMG-1@PMMA surface, we cannot draw any definitive conclusion about the mechanism of action of the POM-IL@PMMA films. Based on our initial findings, we hypothesize that embedding POM-ILs in PMMA could act to reduce the cytotoxicity of the **DOTMG-1**, but further experiments will be required to confirm if hybrid POM-IL@PMMA films do indeed present lower cytotoxicity than the parent POM-IL.

## 4 Conclusions

The sterically demanding cation of the bio-based family of alkyl-guanidinium cations, DOTMG, combined with the lacunary Keggin POM [α-SiW_11_O_39_]^8–^, formed an ionic liquid, **DOTMG-1**, which exhibits broad-spectrum antimicrobial properties at low concentrations. The **DOTMG-1** POM-IL was found to be highly effective against different bacterial strains, including nonpathogenic *E. coli* DH5α and *B. subtilis* as well as pathogenic *L. monocytogenes* and VTEC. The compound possessed higher antibacterial activity against the Gram-positive strains *B. subtilis* and *L. monocytogenes* compared with the Gram-negative strains *E. coli* strains, suggesting that the mechanism of action is linked to the structure of the bacterial cell wall. Importantly, **DOTMG-1** provided a 100% reduction of bacterial colonization of surfaces and prevents subsequent biofilm formation of the same model bacterial strains. Pathogenic molds are reemerging as an increasingly serious threat to human health globally and **DOTMG-1** also possessed high antifungal activity against the model mold strains *A. niger*, *A. ochraceus*, *C. cladosporioides,* and *P. expansum*.

As a proof of concept of the general applicability of the antimicrobial guanidinium POM-IL, the fabrication of multifunctional films showed that such composite materials could be further developed to produce polymeric antimicrobial layers for different surfaces. The **DOTMG-1** POM-IL was embedded in a PMMA polymer matrix and morphological changes in the surface of the films changed with respect to the amount of POM-IL incorporated in the matrix. Antibacterial assays on the films showed that the microbiocidal **DOTMG-1** coating completely prevents bacterial growth and biofilm formation, even at the lowest ratio of **DOTMG-1**/PMMA of 20:80. The results of this work endorse a number of potential future directions for these hybrid materials in the development and implementation of biocompatible antimicrobial surfaces for preventing undesired microbial adhesion.

## Supporting Information Available

NMR spectrum of the POM-IL **DOTMG-1**; characterization of the **DOTMG-1** PMMA films; cytotoxicity of the POM-IL **DOTMG-1**; and data from additional antibacterial assays (PDF)

## Abbreviations

CFU: colony-forming units
DOTMG: *N*,*N*,*N*′,*N*′-tetramethyl-*N*″,*N*″-dioctylguanidinum
POM: polyoxometalate
POM-IL: polyoxometalate-ionic liquid
PMMA: poly(methyl methacrylate)
MIC: minimum inhibitory concentration
MBC: minimum bactericidal concentration
