# Supplementary material for: Hybrid Antimicrobial Films Containing a Polyoxometalate-Ionic Liquid
Source: ACS Appl Polym Mater. 2022 Apr 12;4(6):4144–53. doi: 10.1021/acsapm.2c00110 (PMC9194901; doi:10.1021/acsapm.2c00110)
Supplement: Supplementary file 1 — ap2c00110_si_001.pdf [file ap2c00110_si_001.pdf]

Supporting Information

Hybrid antimicrobial films containing a polyoxometalate-ionic liquid

Ana G. Enderle,†[a,b,c] Isabel Franco-Castillo,†[d,e] Elena Atrián-Blasco,[d,e] Rafael Martín-Rapún,[d,e] Leonardo Lizarraga,[b] María J. Culzoni,[c] Mariela Bollini,[b] Jesús M. de la Fuente,[d,e] Filomena Silva,[f,g] Carsten Streb[a] and Scott G. Mitchell*[d,e]*

a. Institute of Inorganic Chemistry I, Ulm University, Albert-Einstein-Allee 11, 89081 Ulm, Germany.

*Email: carsten.streb@uni-ulm.de

b. Centro de Investigaciones en Bionanociencias (CIBION), CONICET, Godoy Cruz, 2390, C1425FQD Ciudad de Buenos Aires, Argentina.

c. Laboratorio de Desarrollo Analítico y Quimiometría (LADAQ), Universidad Nacional del Litoral – CONICET, Ciudad Universitaria, Paraje El Pozo, CC242, S3000 Santa Fe, Argentina

d. Instituto de Nanociencia y Materiales de Aragón (INMA-CSIC), Consejo Superior de Investigaciones Científicas-Universidad de Zaragoza, c/ Pedro Cerbuna 12, 50009 Zaragoza, Spain.

e. CIBER de Bioingeniería, Biomateriales y Nanomedicina, Instituto de Salud Carlos III, 28029 Madrid, Spain.

*Email: scott.mitchell@csic.es

f. ARAID – Agencia Aragonesa para la Investigación y el Desarrollo, Av. Ranillas, 1D, 2B, 50018 Zaragoza, Spain.

g. Universidad de Zaragoza, Facultad de Veterinaria, Calle Miguel Servet 117, 50013 Zaragoza, Spain.

† These authors have contributed equally to this publication.

Supporting Information

Table of Contents

1. NMR spectrum of the POM-IL DOTMG-1 **S3**

2. TGA of POM-IL DOTMG-1 **S6**

3. Preparation and characterization of the DOTMG-1 PMMA films **S7**

4. Cytotoxicity of the POM-IL DOTMG-1 **S12**

5. Antibacterial Studies **S13**

1. NMR spectrum of the POM-IL

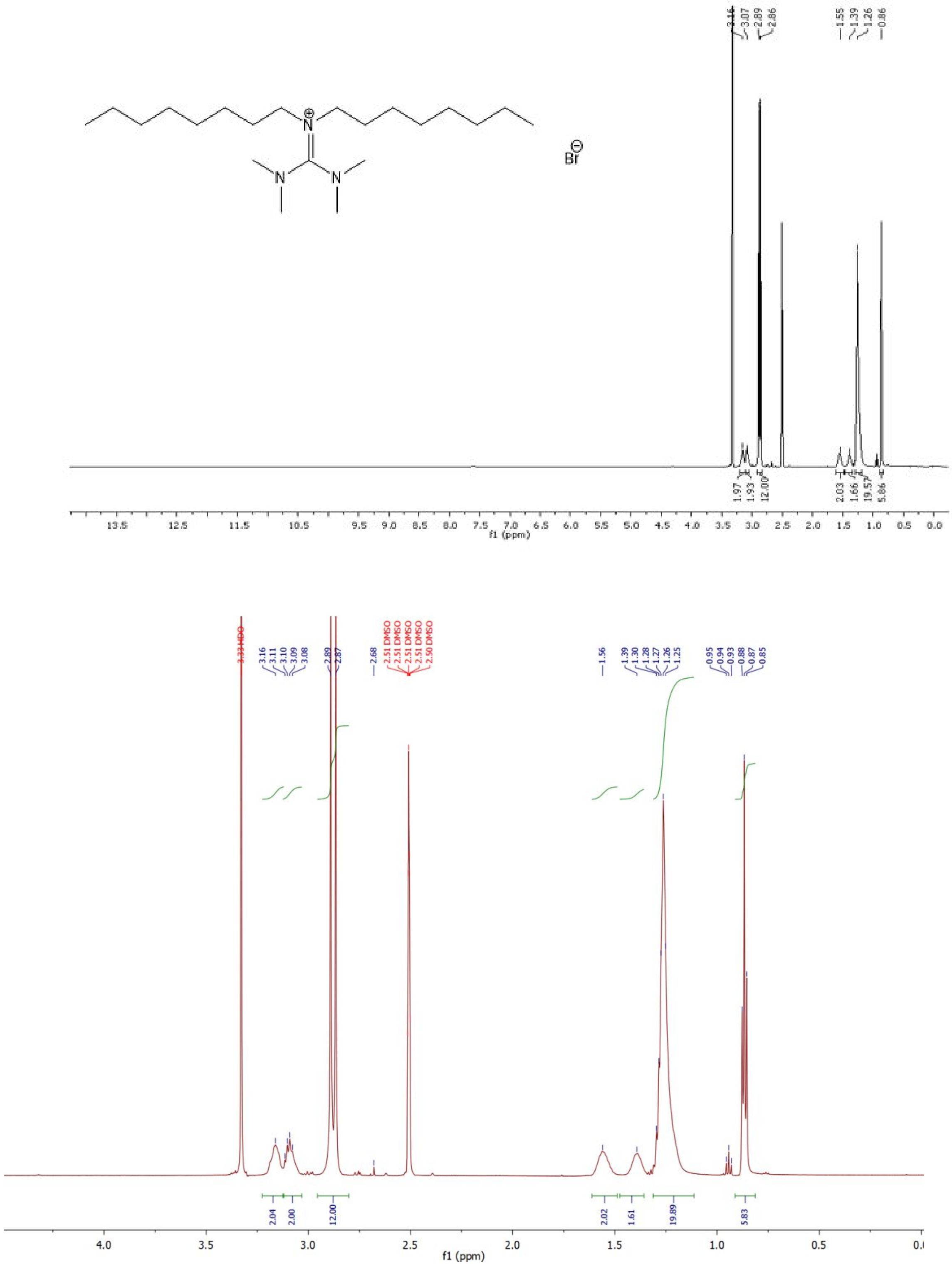

Figure S1. ^{1}H-NMR spectrum of DOTMG-Br in DMSO-d6.

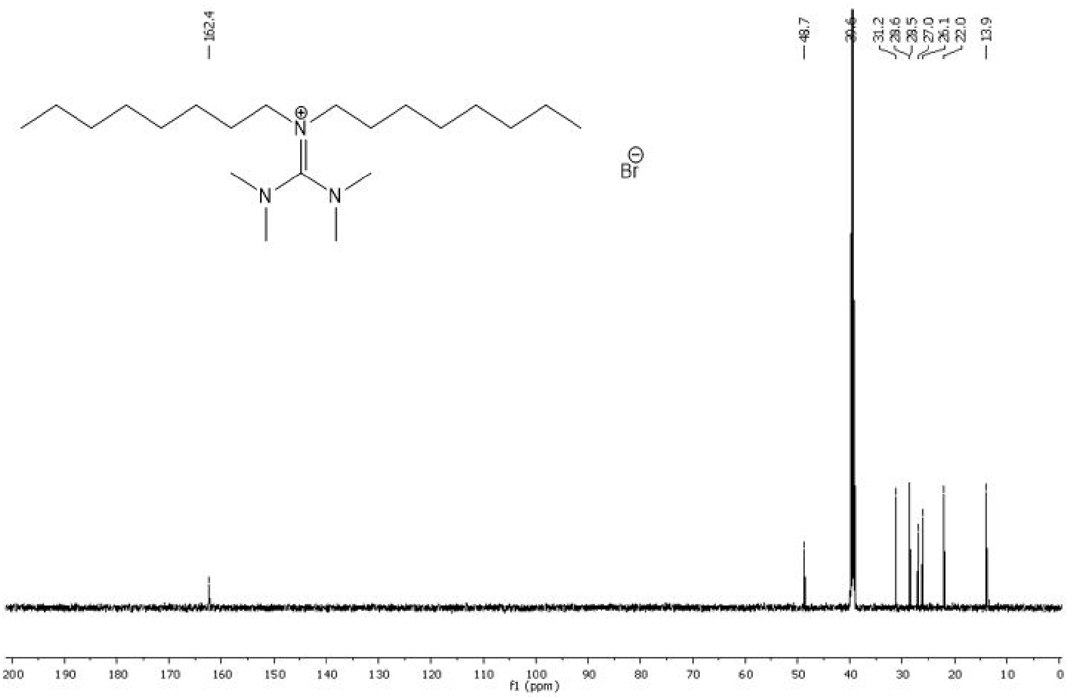

Figure S2. ^{13}C-NMR spectrum of DOTMG-Br in DMSO-d6.

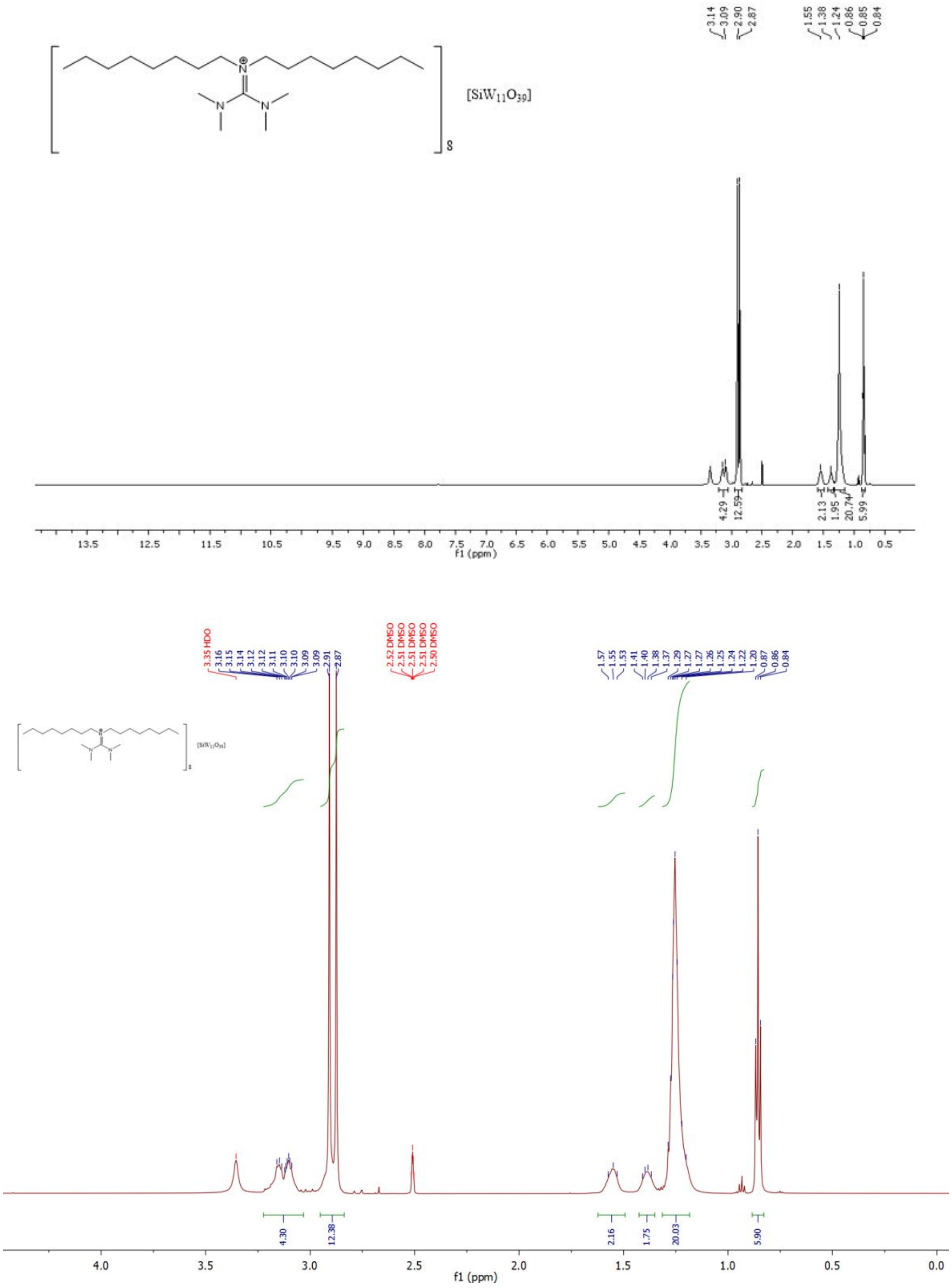

Figure S3. ^{1}H-NMR spectrum of DOTMG-1 in DMSO-d6.

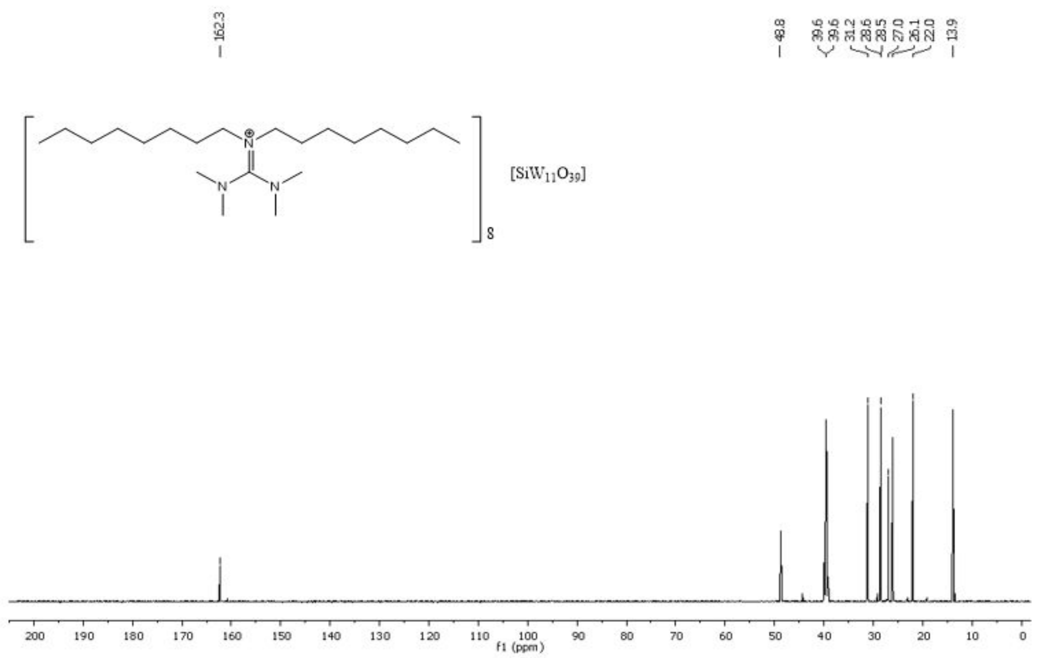

Figure S4. ^{13}C-NMR spectrum of DOTMG-1 in DMSO-d6.

2. TGA graph of the POM-IL DOTMG-1

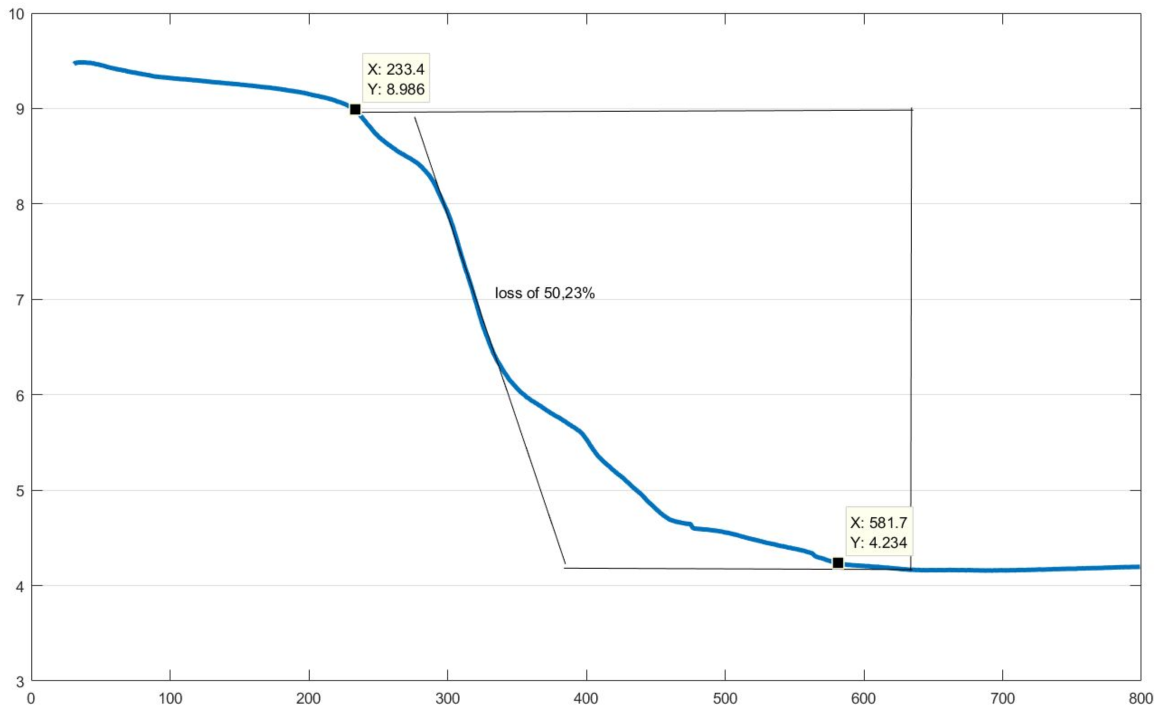

Figure S5. TGA graph of DOTMG-1.

3. Preparation and characterization of the DOTMG-1 PMMA films

Table S1. Composition of the films

Film	DOTMG-1 (mg)	Toluene (mL)	Solution A (mL)
**B**	4	0.04	0.16
**C**	7	0.07	0.13
**D**	10	0.10	0.10

- **FT-IR analysis**

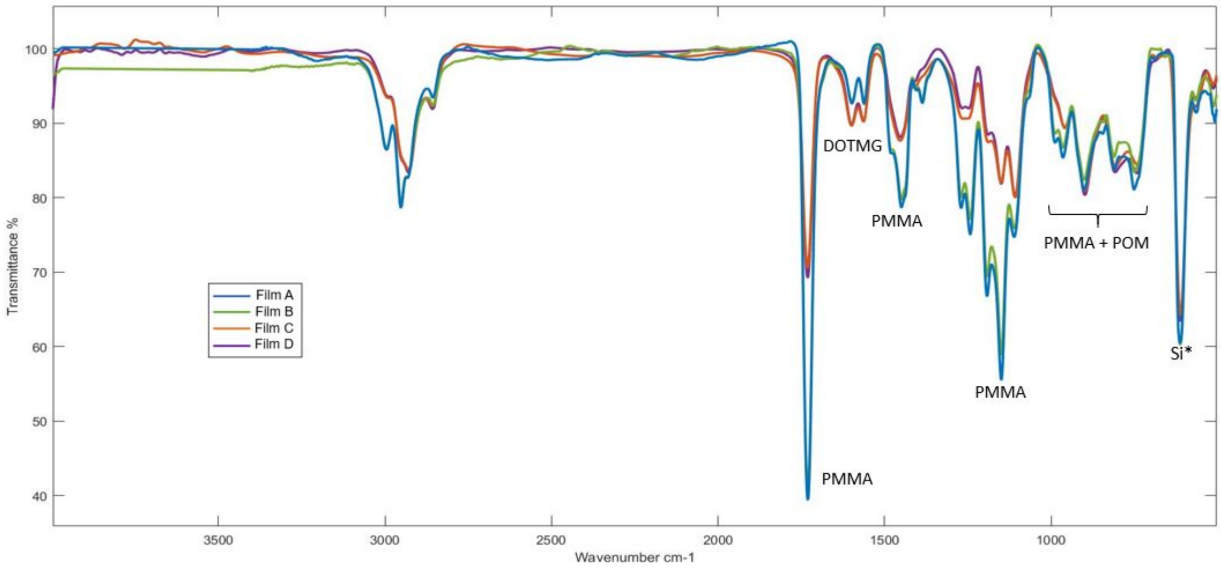

Figure S6. FT-IR analysis of films A, B, C and D.

It is possible to visualize the changes in the signals corresponding to PMMA and DOTMG-1 when increasing the POM-IL content on the films from B to C. The peaks at 610 cm−1 and 1115 cm-1 correspond to the Si lattice phonons and to Si-O2 complexes, respectively.32 The main peaks of PMMA are observed at 1713 cm−1, 1425 cm−1, 1125 cm−1 and in the region between 1000 to 750 cm−1.33 It can be observed that the region corresponding to the 750-1000 cm−1 is an overlapping zone of POM and PMMA signals. Two peaks corresponding to the cation in 1603 cm−1 and 1562 cm−1 are more visible on the spectra corresponding to films C and D.SEM studies

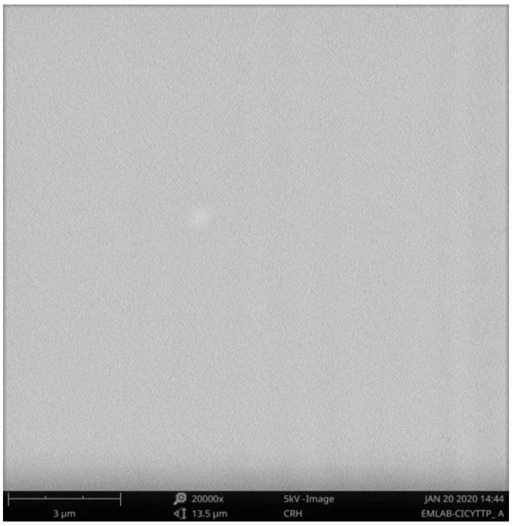

Figure S7. SEM micrographs of Film A. Scale bar 3 µm.

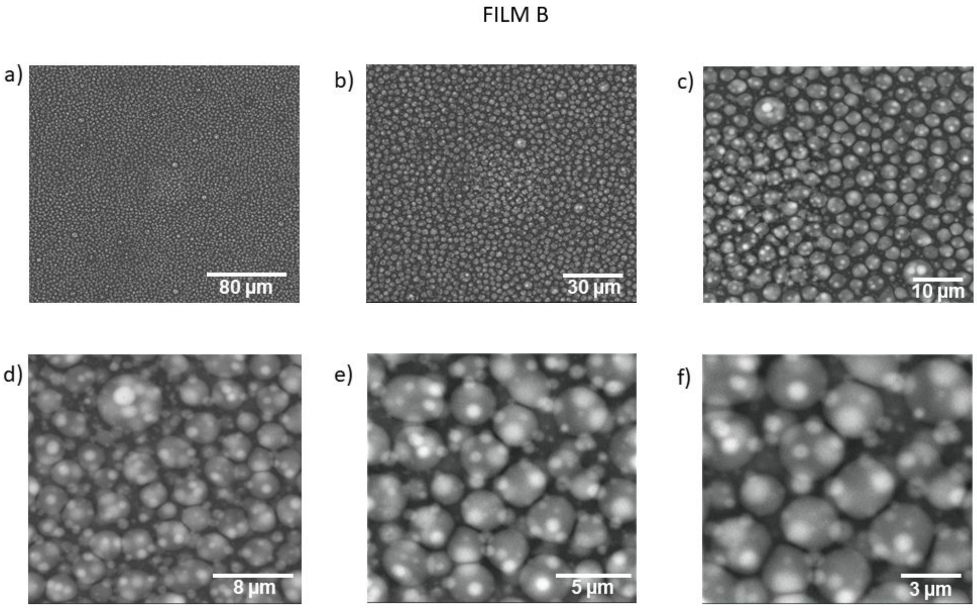

Figure S8. SEM micrographs of film B. Magnifications: a) 1000x; b) 2000x; c) 5000x; d) 10000; e) 15000x; f) 20000x

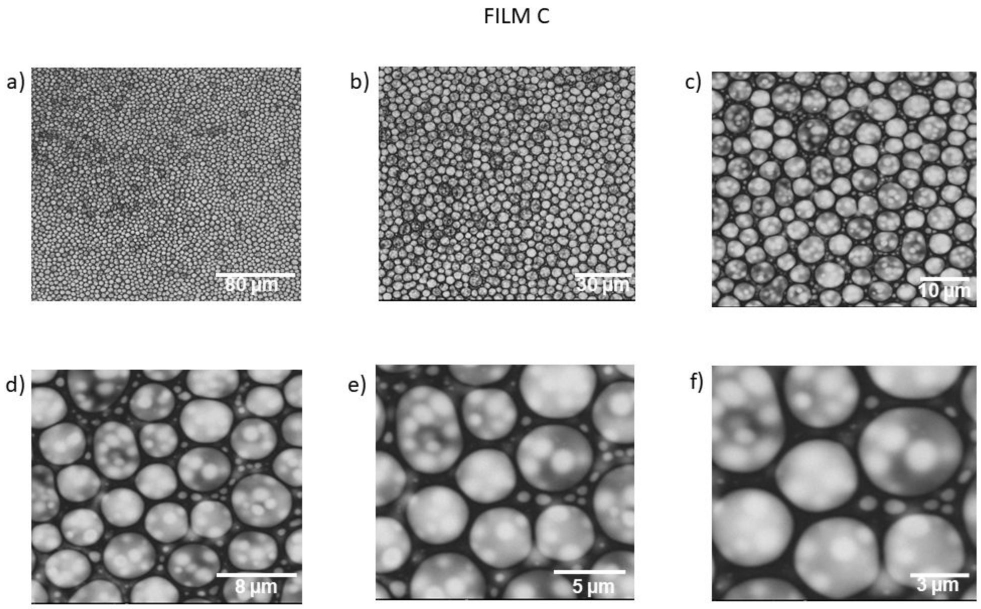

Figure S9. SEM micrographs of film C. Magnifications: a) 1000x; b) 2000x; c) 5000x; d) 10000; e) 15000x; f) 20000x.

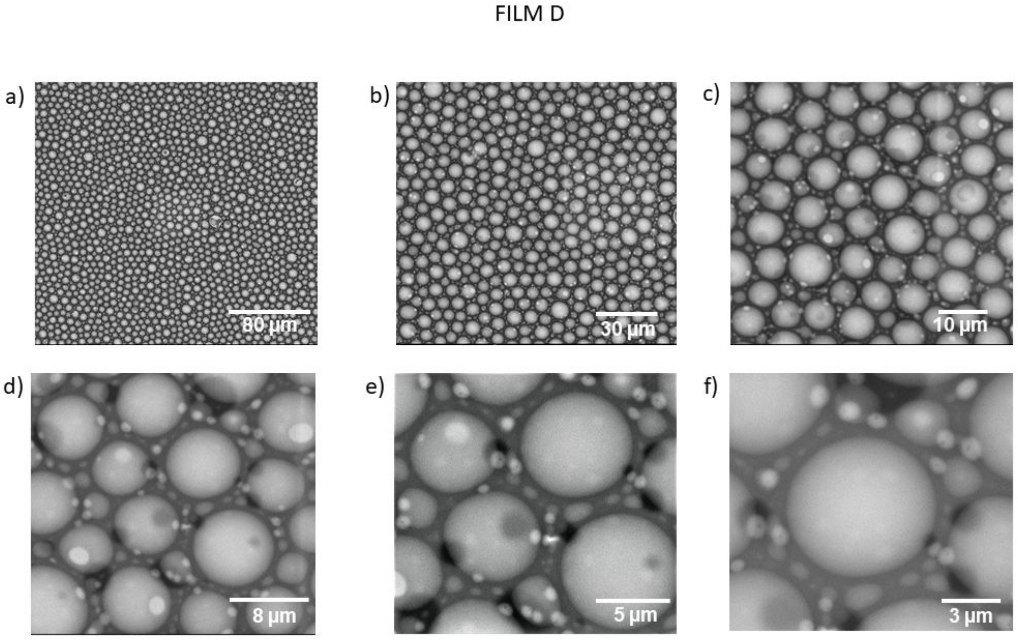

Figure S10. SEM micrographs of film D. Magnifications: a) 1000x; b) 2000x; c) 5000x; d) 10000; e) 15000x; f) 20000x.

Supporting Information

AFM images

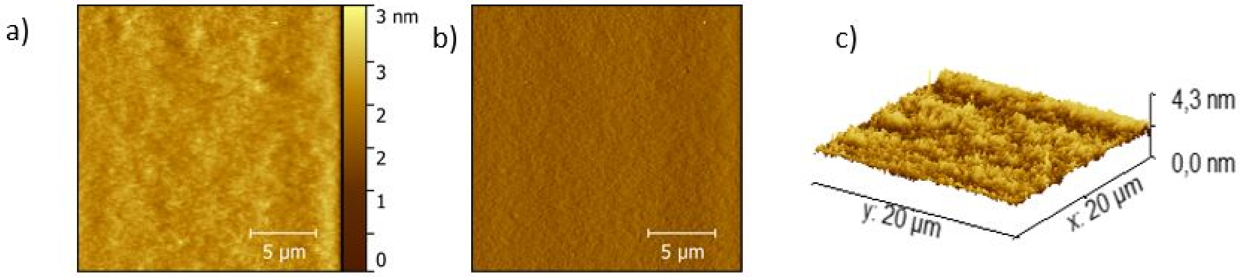

Figure S11. AFM image of Film A, a) Height image, b) phase image, and c) 3D height image.

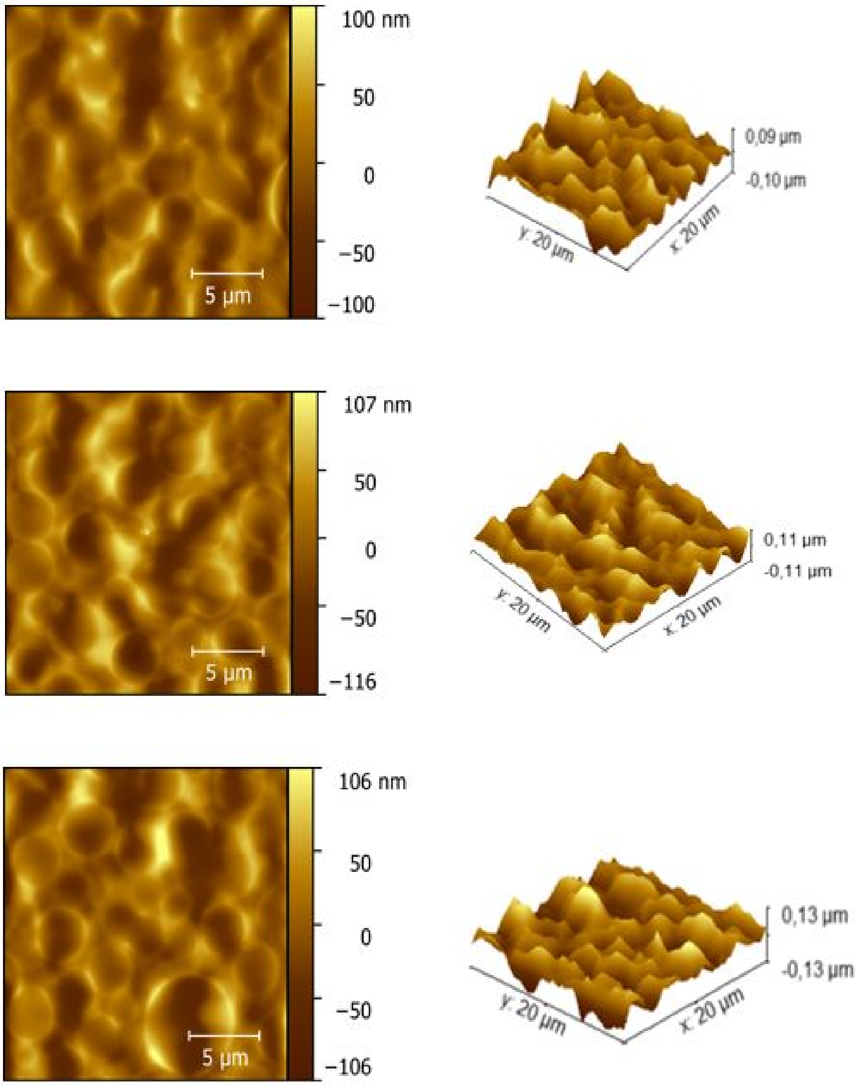

Figure S12. AFM images of Film B; Left: Height images; right: 3D height images (different positions).

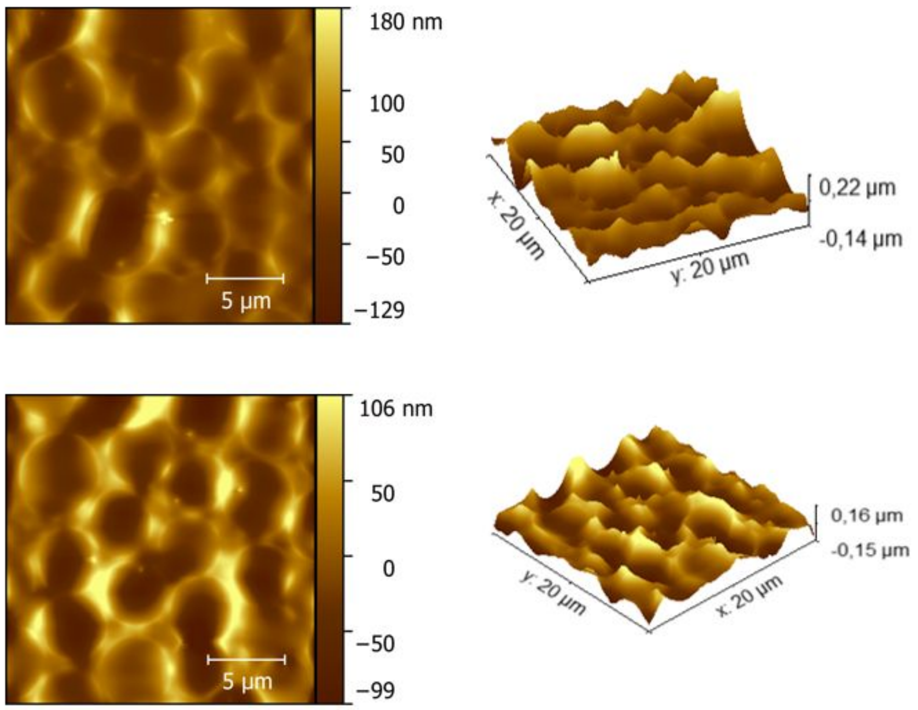

Figure S13. AFM images of Film C; Left: Height images; right: 3D height images (different positions).

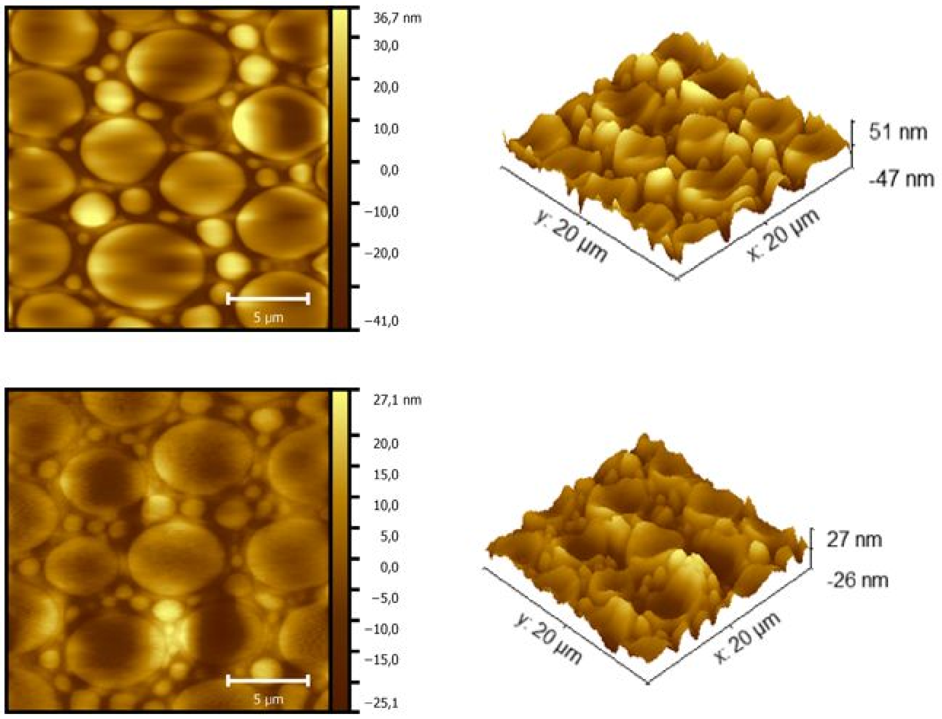

Figure S14. AFM images of Film D; Left: height images; right: 3D height images (different positions).

Supporting Information

From the height profile of the samples studied by AFM, it is possible to calculate the surface roughness. In film D, the droplets are larger, but the surface is flatter, and the roughness decreases compared with films C and B. From the comparison of the roughness of B and C, it is observed that this value increases when the content of the IL increases (Table S2).

Table S2. Root mean square roughness of the films (Sq)

Film	Sq (nm)
A	0.4
B	33.8 +/- 0.1
C	36.7 +/- 0.2
D	9.4 +/- 0.1

4. Cytotoxicity of the POM-IL DOTMG-1

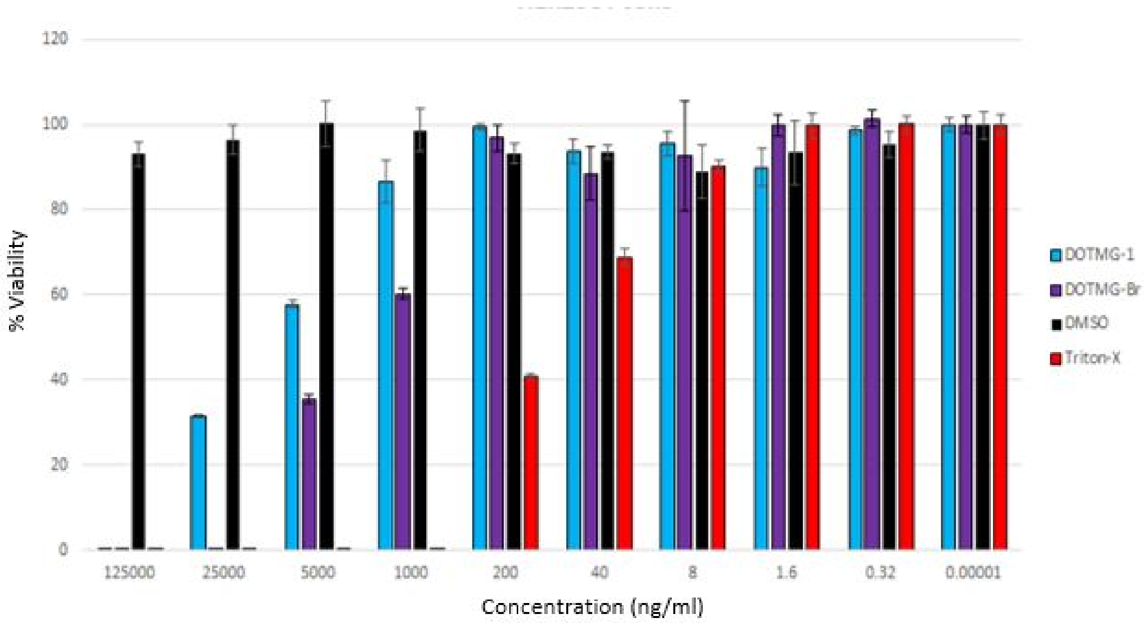

Figure S15. Concentration dependent *in vitro* cytotoxic effect of DOTMG-1 and DOTMG-Br on the viability of HEK293T cells.

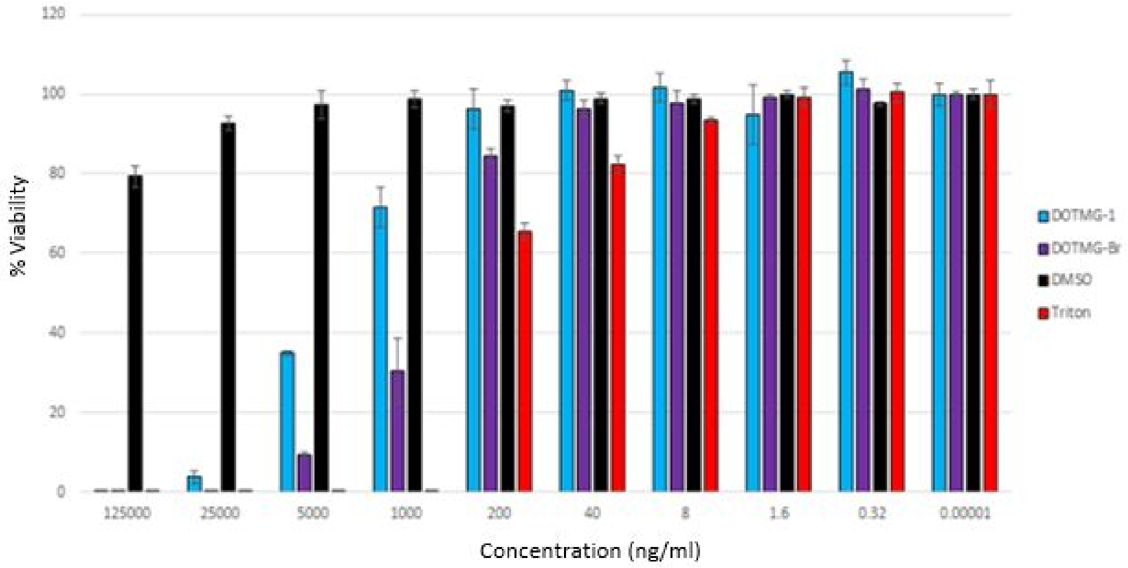

Figure S16. Concentration dependent *in vitro* cytotoxic effect of DOTMG-1 and DOTMG-Br on the viability of TZM-bl cells.

5. Antibacterial Studies

Table S3. Microorganisms and growth conditions.

Microorganisms	Solid media	Liquid media	Incubation Temperature	Inoculum incubation
**Bacteria**				
E. coli DH5α	TSA	LB	37 ^{0}C	24 hours
VTEC	MHA	LB	37 ^{0}C	24 hours
B. subtilis	TSA	NB	37 ^{0}C	24 hours
L. monocytogenes	BHIA	NB	37 ^{0}C	24 hours
**Fungi**				
A. niger	SDA	RPMI	35 ^{0}C	4 days
A. ochraceus	YMA	MEP	25 ^{0}C	4 days
C. cladosporioides	SDA	RPMI	25 ^{0}C	4 days
P. expansum	PDA	YMB	25 ^{0}C	4 days

Supporting Information

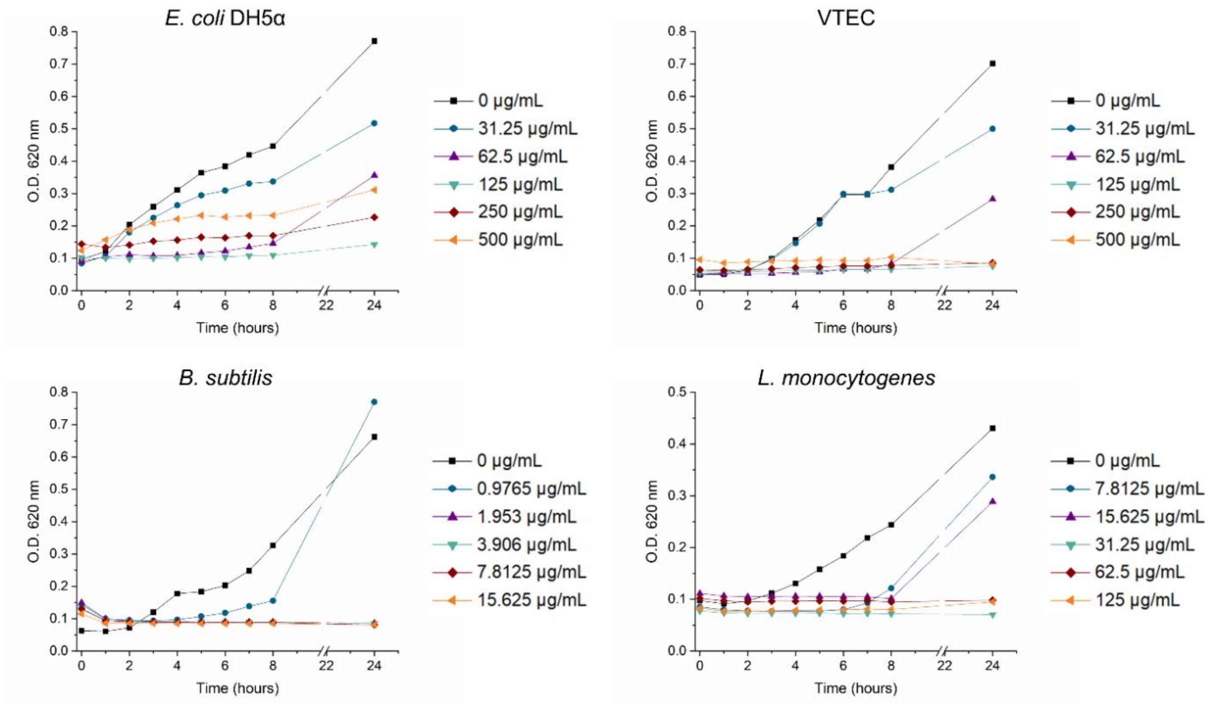

Figure S17. *E. coli* DH5α, VTEC, *B. subtilis* and *L. monocytogenes* growth curves, starting with a 10^7 CFU/mL inoculum, in the presence of different DOTMG-1 concentrations (ranging from 0.9765 to 500 µg/mL). The minimum inhibitory concentration (MIC) obtained for the different bacteria was: 125 µg/mL for *E. coli* DH5α and VTEC; 31.25 µg/mL for *L. monocytogenes* and 1.95 µg/mL for *B. subtilis*. The apparent increase of turbidity in the *E. coli* DH5α graph at the higher concentrations is due to the precipitation of the compound in the culture media. These results were confirmed with the Resaruzin assay and by subculturing in solid culture media, as described in section 3.5.1.2. Bacterial cell viability assay.

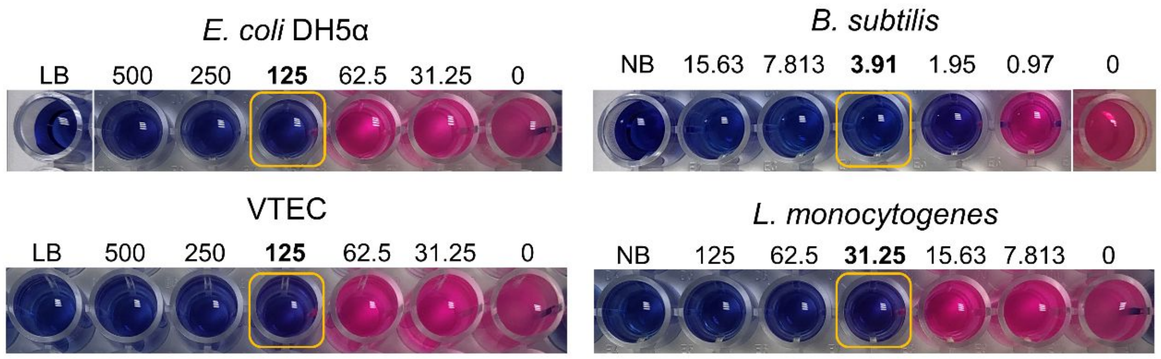

Figure S18. Resazurin cell viability assay for *E. coli* DH5α, VTEC, *B. subtilis* and *L. monocytogenes*, incubated with different concentrations of DOTMG-1 (in µg/mL). The Resazurin compound (blue) turns pink in presence of viable bacteria as a result of their metabolic activity, therefore pink wells indicate alive bacteria, while blue wells indicate a loss of metabolic activity, which is one of the first cascade events in the mechanism of cell death.

The results were confirmed by subculturing on solid media and colony counting. Hence, the minimum bactericidal concentration (MBC) for both *E. coli* (*E. coli* DH5α and VTEC) is 125 μg/mL, 3.91 μg/mL for *B. subtilis* and 31.35 μg/mL for *L. monocytogenes*. The MBC values of both *E. coli* strains and *L. monocytogenes* agree with the MIC value, while *B. subtilis* presents a MBC value twice that of the corresponding MIC.

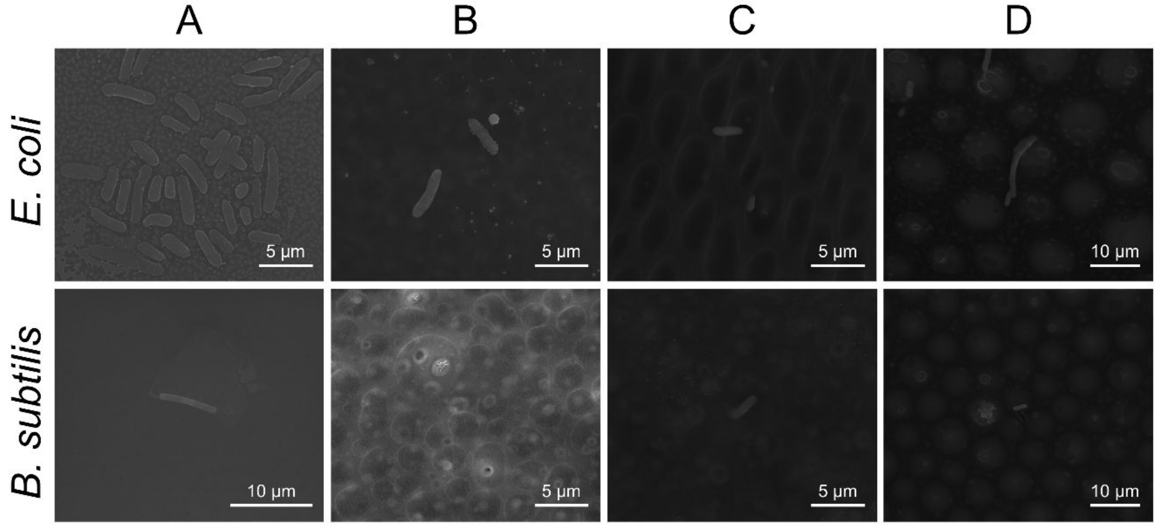

Figure S19. ESEM images of the DOTMG-1@PMMA films after incubation with *E. coli* and *B. subtilis*. All the samples were rinsed after the incubation time in order to sow the planktonic bacteria and determine the antimicrobial performance of the films. The PMMA film without DOTMG-1 (A) presented regular *E. coli* growth over the surface and the bacteria exhibited good morphology. Very few bacteria were found on the surface of the DOTMG-1@PMMA films (B, C, and D). In the case of *B. subtilis*, hardly any bacteria were found, even in the films without POM-IL (A), probably due to low adhesion of the bacterial cells to this surface of the PMMA.
